# Prenatal antidepressant exposure and emotional disorders until age 22: a danish register study

**DOI:** 10.1186/s13034-023-00624-9

**Published:** 2023-06-16

**Authors:** Mette Bliddal, Rikke Wesselhoeft, Katrine Strandberg-Larsen, Martin T. Ernst, Myrna M. Weissman, Jay A. Gingrich, Ardesheer Talati, Anton Pottegård

**Affiliations:** 1 Research unit OPEN, Department of Clinical Research, University of Southern Denmark, Odense, Denmark; 2 Department of Clinical Pharmacology, Pharmacy and Environmental Medicine, University of Southern Denmark, Odense, Denmark; 3 Department of Child and Adolescent Psychiatry, Odense Mental Health Services in the Region of Southern Denmark, Odense, Denmark; 4 Section of Epidemiology, Department of Public Health, University of Copenhagen, Copenhagen, Denmark; 5 Department of Psychiatry, Columbia University Irving Medical Center and Vagelos College of Physicians and Surgeons, New York, NY USA; 6 New York State Psychiatric Institute, New York, NY USA; 7 Mailman School of Public Health, Columbia University, New York, NY USA; 8 Columbia University and New York State Psychiatric Institute, 1051 Riverside Drive, Suite 6402B / Unit 24, New York, NY 10032 USA

**Keywords:** Depression, Antidepressant, Selective serotonin reuptake inhibitor (SSRI), Prenatal exposure, Birth cohort, Propensity score

## Abstract

**Background:**

Selective serotonin reuptake inhibitors (SSRIs) are the most frequently prescribed antidepressants in pregnancy. Animal and some clinical studies have suggested potential increases in depression and anxiety following prenatal SSRI exposure, but the extent to which these are driven by the medication remains unclear. We used Danish population data to test associations between maternal SSRI use during pregnancy and children outcomes up to age 22.

**Methods:**

We prospectively followed 1,094,202 single-birth Danish children born 1997–2015. The primary exposure was ≥ 1 SSRI prescription filled during pregnancy; the primary outcome, first diagnosis of a depressive, anxiety, or adjustment disorder, or redeemed prescription for an antidepressant medication. We used propensity score weights to adjust potential confounders, and incorporated data from the Danish National Birth Cohort (1997–2003) to further quantify potential residual confounding by subclinical factors.

**Results:**

The final dataset included 15,651 exposed and 896,818 unexposed, children. After adjustments, SSRI-exposed had higher rates of the primary outcome than those of mothers who either did not use an SSRI (HR = 1.55 [95%CI:1.44,1.67] or discontinued the SSRI use ≥ 3 months prior to conception (HR = 1.23 [1.13,1.34]). Age of onset was earlier among exposed (9 [IQR:7–13] years) versus unexposed (12 [IQR:12–17] years) children (p < 0.01). Paternal SSRI use in the absence of maternal use during the index pregnancy (HR = 1.46 [1.35,1.58]) and maternal SSRI use only after pregnancy (HR = 1.42 [1.35,1.49]) were each also associated with these outcomes.

**Conclusions:**

While SSRI exposure was associated with increased risk in the children, this risk may be driven at least partly by underlying severity of maternal illness or other confounding factors.

**Supplementary Information:**

The online version contains supplementary material available at 10.1186/s13034-023-00624-9.

## Introduction

Major depressive disorder is a psychiatric disorder associated with significant distress and functional impairment worldwide. Increasing rates of depression during pregnancy have prompted the US preventative services task force to recommend routine depression screening for women of childbearing age [1]. Selective serotonin reuptake inhibitors (SSRIs) are the most frequently prescribed class of antidepressant medications in pregnancy; hence, SSRI use has also increased over the last two decades [2–5]. SSRIs are often effective for mitigating maternal depression, but they readily cross the placenta and fetal blood-brain barrier [6]. SSRI medications have been shown to impact brain development in preclinical models, leading to potential concern about their effects on human fetal neurodevelopment when used in pregnancy [7, 8]. Addressing these concerns through observational research presents a challenge: as the effects of SSRI exposure may be confounded by child exposure to maternal depression.

Much of the initial literature studying the effects of SSRI exposure had focused on very early endpoints such as neonatal and early life outcomes [9–12]. However, pre-clinical studies by us and others suggested that early-life SSRI exposures affect brain development in critical brain regions (prefrontal cortex, amygdala, hippocampus) that ultimately predispose SSRI-exposed animals to augmented rates of depression and anxiety-like behaviors that only emerge in the peri-adolescence period [13, 14]. Initially translating these findings to humans, we found that children prenatally exposed to SSRIs had higher risk (hazard ratio, *HR* = 1.78) for depressive disorders than their unexposed counterparts [15]. This increased risk did not begin to emerge until age 10, and in that study peaked around age 14, when follow-up ended. Some other studies have also reported increases in teen affective disorders as a function of prenatal SSRI exposure,[16–19] but no study has followed children into adulthood, or fully disentangled the extent to which these associations may reflect pharmacological effects of the medication versus the underlying maternal illness – a potentially important confounder that animal studies do not capture [18]

Here, we examine the effects of SSRI use in pregnancy on both diagnoses and medication treatment relating to child and adolescent emotional (depression, anxiety, and adjustment) disorders using Danish nationwide register data. To address limitations in previous research, we [1] use propensity score-weighted and matched approaches to adjust for potential confounding variables; and [2] leverage prospectively collected data from Danish National Birth Cohort (DNBC)[20] within the larger register data to test for sub-clinical differences that are typically not quantifiable from register data alone. Finally [3], we follow children up to age 22, traversing a greater period of risk for the onset of emotional disorders than previous studies, and making this the longest follow-up study of gestational SSRI exposures.

## Method

We created a cohort from Danish national health registers, and within that, a nested sample from the Danish National Birth Cohort (DNBC). The study unit was the individual pregnancy/child, and the analyses were designed to consider the underlying mental health of the mother. We included singleton children born from January 1, 1997, through December 31, 2015. The institutional data protection board at the University of Southern Denmark, the Danish Health Data Authority, and the DNBC approved the research project. As per Danish law, studies based entirely on registry data do not require approval from an ethics review board.

### Setting and data sources

Danish healthcare services are free, and each healthcare contact is monitored at the individual level in administrative registers. Linkage of individual records across registers are ensured by a unique personal identification number assigned to all Danish inhabitants at birth or first immigration [21]. Maternal information was obtained through the Danish National Patient Register [22] the Danish Medical Birth Register [23], the Danish Prescription Register [24], and the Danish Civil Register [21] (details in Supplementary Material). Information on psychiatric disorders was retrieved from the Danish National Patient Register, based on the Danish version of the International Classification of Diseases, 10th revision (ICD-10). Additional information on maternal socio-demographics was obtained from the Civil Person Registration system, the Income Statistics Register,[25] and the Danish Education Register [26]. We obtained information on sub-clinical indicators such as self-reported alcohol use, planning of pregnancy and self-reported psychopathology from the DNBC sub-cohort (the DNBC only included births from 1997 to 2003 [20]; Supplementary Material).

### Cohorts

**Fig. 1:**
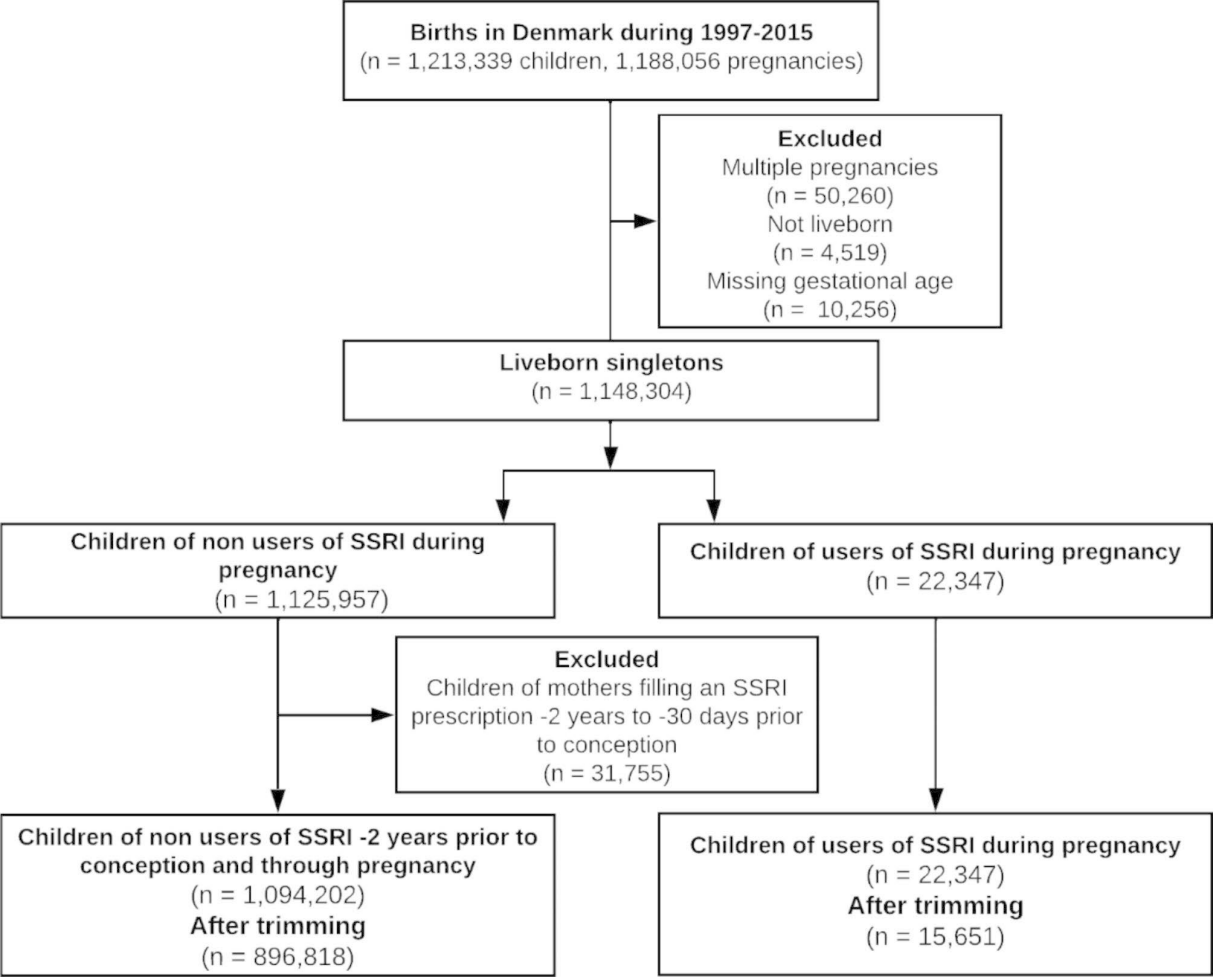
Flow chart of the study population based on children born in Denmark 1997–2015

The Danish Medical Birth Register was used to identify children born 1997–2015. We excluded multiple deliveries and pregnancies with missing information on gestational age (Fig. 1). The exposed cohort consisted of pregnancies where mothers redeemed any prescriptions on SSRIs (ATC code N06AB) from 30 days prior to conception (which was defined as day of delivery minus gestational age) to the day of delivery. The unexposed cohort included pregnancies with no redeemed SSRI prescriptions from 2 years before conception until day of delivery. Pregnancies that did not meet either criterion were not included in either cohort, to minimize misclassification bias.

### Outcomes

Outcomes included any diagnosis of a depressive disorder, anxiety disorder, or adjustment disorders, and/or redeeming any prescriptions of antidepressants, including SSRIs, tricyclic antidepressants (TCAs), or ‘other antidepressants’ (see Table S1). While each were also analyzed separately, the main outcome was a composite measure comprising all these categories, that is, first diagnosis or redeemed drug, whichever came first.

### Main analysis

We used propensity score (PS) methods to adjust for potential confounding [27]. We estimated the propensity for SSRI exposure, i.e., the individual’s PS, for each person based on the covariates specified in Supplementary Material from the National registers using logistic regression. To reduce bias further, we trimmed our cohorts as suggested by Stürmer et al [28] in order to exclude women treated or untreated contrary to prediction. Hence, we obtained a propensity score that was common to both exposed and unexposed by trimming the lower and upper PS with 1%. Balance between the cohorts was achieved using standardized mortality weighting [29]. Missing information was limited (< 4%) except for data on body mass index and smoking which was not registered systematically in the early study period. For PS, missing variables were coded as “missing”, that is, a separate category.

The study was originally planned using PS matching, but due to exclusion of many exposed individuals using this method, we changed the main analyses to standardized mortality weighting. The main results were, however, also calculated using propensity score matching. For each pregnancy in the SSRI exposed cohort, we matched 8 pregnancies from the unexposed cohort using propensity score nearest-neighbor matching [30] in order to match exposed and unexposed with the same probability of being exposed (max caliper 0.01). This model was constructed in three calendar year strata (1997–2003, 2004–2009, 2010–2015) to account for time varying indications or changes in clinical preferences.

We calculated standard mean differences (*SMD*s) between exposed and unexposed children to examine comparability. We followed each child from their third birthday to first event of a diagnosis or filled prescription, emigration, death, or end of follow-up (December 31st, 2018). We used Cox regression analysis to calculate hazard ratios with 95% confidence intervals (95% CI), with child age as the time scale. Cluster methods were used to account for dependency between siblings. To illustrate the cumulative incidence of the main outcome, we plotted Kaplan-Meier curves for exposed and unexposed groups by age at first diagnosis/first prescription.

### Sensitivity analyses

To test the robustness of our findings and control for confounding by indication and severity, we completed the following supplementary analyses. To test whether mothers using SSRIs differed on subclinical indicators of depression compared to those not using SSRIs, we compared exposed and PS-weighted unexposed women from the DNBC subsample according to the sub-clinical self-reported covariates described in the supplementary material. To **quantify effects of SSRI exposure beyond those of maternal illness exposure**, we constructed the exposed and unexposed groups only among women with a documented psychiatric disorder within one year prior to pregnancy (ICD-10 F00-F99). To **address confounding by indication**, we compared women who used an SSRI during pregnancy to those who used an SSRI between 2 years and 30 days prior to conception, but not during pregnancy. To **reduce misclassification resulting from women redeeming but not taking SSRIs**, we repeated the analysis redefining users as women who filled ≥ 2 prescriptions during the exposure window. To **address the timing of exposures**, we tested exposure recorded in each trimester separately. To **rule out effects of severe psychopathology**, we excluded women who filled a prescription of antipsychotics (Anatomical Therapeutic Chemical [ATC] classification code N05*) during pregnancy (30 days before conception to birth date). To **test specificity of exposure** to the medication during the prenatal period, we examined (a) the associations between the father’s SSRI use during the mother’s corresponding pregnancy and children outcomes, and (b) effects of maternal SSRI use in the first 24 months of life but not during pregnancy. We hypothesized that if the associations between maternal SSRI use and child outcomes are being driven by an intrauterine pharmacological mechanism, then paternal use or maternal postnatal use should not be associated with the same outcomes.

Finally, we created a combined model including seven mutually exclusive groups: (1) maternal prenatal exposure only, (2) maternal postpartum exposure only, (3) prenatal paternal exposure only, (4) maternal pre- and postnatal exposure, (5) maternal and paternal prenatal exposure, (6) paternal prenatal and maternal postnatal exposure, and (7) maternal and paternal prenatal and maternal postnatal exposure (the reference group was no maternal or paternal exposure either pre- or postnatal).

## Results

### Baseline characteristics

Of 1,213,339 children born in Denmark during 1997–2015, 1,116,549 (92%) met all inclusion criteria as detailed in Fig. 1. Of these, 22,347 (2.04%) met criteria for prenatal exposure to SSRIs. Following trimming, the final analytic dataset included 15,651 exposed, and 896,818 unexposed, children. Of the mothers on an SSRI in pregnancy, 95.6% were prescribed SSRI monotherapy; 4.4% were prescribed more than one psychotropic medication.

Mothers using SSRIs were more likely to be overweight, smoke, be unemployed, and have fewer years of education (Table 1, left). Propensity-score (PS)-weighting reduced these group differences (SMD < 0.1, right-most columns of Table 1) except for some psychiatric indicators (e.g., visiting private psychiatrists, or filling other prescriptions, **Table S2**). For the subset of mothers with additional sub-clinical data available from the Danish National Birth Cohort, PS-weighting using register information also reduced group differences for many subclinical factors, but differences remained for self-reported psychopathology, planned pregnancy, and suffering from eating disorders (SMD > 0.20) (**Table S3**).

**Table 1:** Characteristics of the study cohort according to SSRI exposure during pregnancy (1997–2015)

	Exposed	Unexposed	Weighted- Unexposed	SMD*
	(N = 15,651)	(N = 896,816)	(n = 14,959)	
**MOTHERS**	N (%)	N (%)	N (%)	
*Age*				
Median (IQR, years)	30 (27–34)	30 (27–33)	30 (26–34)	0.02
*Parity*				
First childbirth	6742 (43)	388,114 (43)	6,390 (43)	0.01
2–3 childbirth	7770 (50)	460,634 (52)	7,536 (50)	0.01
4 + childbirth	1084 (7.0)	45,665 (5.1)	984 (6.6)	0.01
*Body mass index*				
Underweight	560 (4.6)	25,091 (4.3)	505 (3.4)	0.01
Normal weight	6689 (55)	358,529 (62)	6,447 (43)	0.01
Overweight	2748 (23)	122,910 (21)	2,596 (17)	0.01
Obese, class 1	1282 (11)	46,557 (8.0)	1,185 (7.9)	0.01
Obese, class 2 & 3	866 (7.1)	27,011 (4.7)	788 (5.3)	0.01
*Smoking in pregnancy*				
No smoking	10,897 (73)	706,880 (84)	10,656 (71)	0.04
Light smoking (1–10 cigarettes/day)	2775 (19)	103,016 (12)	2,541 (17)	0.02
Heavy smoking (11 + cigarettes/day)	1259 (8.4)	34,332 (4.1)	1,079 (7.2)	0.03
*Marital status*				
Unmarried	8616 (55)	426,635 (48)	8,055 (54)	0.02
Married/registered partnership	7034 (45)	470,065 (52)	6,904 (46)	0.02
*Employment status*				
Un-employed	6503 (42)	246,077 (27)	5,812 (39)	0.06
Student	940 (6.0)	53,299 (5.9)	925 (6.2)	0.01
Employed	7935 (51)	577,870 (64)	7,947 (53)	0.05
Self-employed	272 (1.7)	19,513 (2.2)	275 (1.8)	0.01
*Highest education*				
Short (7–10 years)	4428 (29)	164,174 (19)	3,919 (26)	0.05
Vocational training	4357 (28)	261,434 (30)	4,292 (28)	0.02
Medium (11–13 years)	2266 (15)	139,282 (16)	2,181 (15)	0.00
Long (13 + years)	4279 (28)	309,088 (35)	4,274 (29)	0.03
*Income*				
First quartile (Lowest)	5318 (34)	203,342 (23)	4,706 (32)	0.05
Second quartile	6227 (40)	342,024 (38)	6,143 (41)	0.03
Third quartile	2934 (19)	235,792 (26)	2,931 (20)	0.02
Forth quartile (Highest)	1159 (7.4)	114,734 (13)	1,168 (7.8)	0.02
**Children**				
*Gender*				
Male	8098 (52)	460,255 (51)	7,676 (51)	0.01
Female	7553 (48)	436,563 (49)	7,283 (49)	0.01
*Birth year*				
1997–2003	3017 (19)	291,736 (33)	2,966 (20)	0.01
2004–2009	6218 (40)	322,830 (36)	6,402 (43)	0.06
2010–2015	6416 (41)	282,252 (31%)	5,591 (37)	0.07

*Standard Mean Difference

### SSRI exposure associated with emotional disorders and antidepressant use in children

**Fig. 2:**
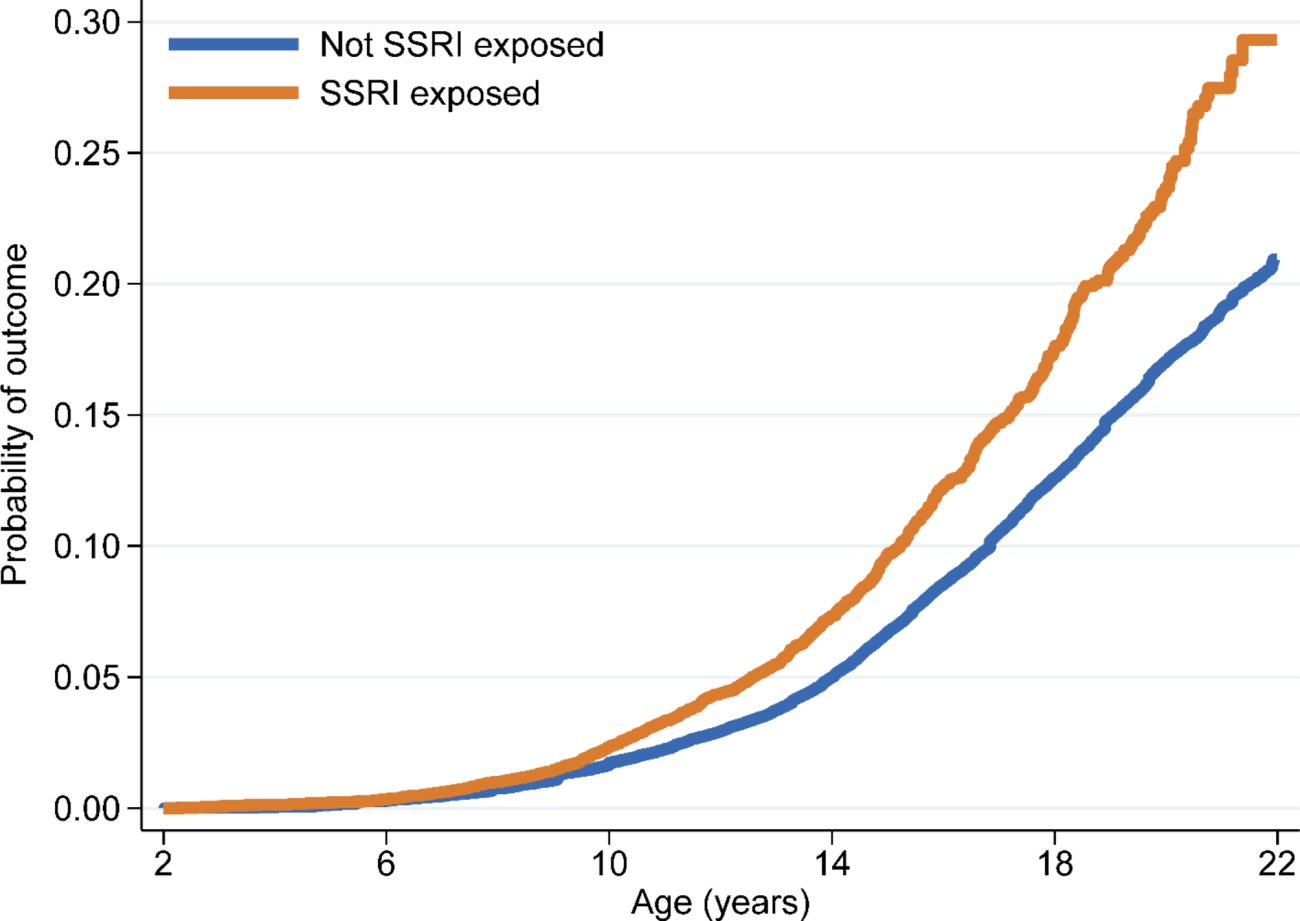
Failure plot on hazard rates for emotional disorders (diagnosis or prescription) in offspring by maternal exposure to selective serotonin reuptake inhibitors (SSRI) during pregnancy in Denmark (1997–2015)

When examined by prenatal SSRI exposure, 4.6% of exposed, versus 3.9% of unexposed, children met criteria for an emotional disorder diagnosis or redeemed an antidepressant prescription (***HR*** = 1.55 [95% CI: 1.44–1.67]; Table 2); the strongest associations were between maternal SSRI use and child SSRI prescriptions (*HR* = 2.18 [1.89–2.52]). The rate differences, illustrated in Fig. 2, show that differences emerge around age 9 and endure through follow-up till age 22. Age of onset was also earlier among exposed (9 years, interquartile range (IQR): 7–13) compared to unexposed (12 years, IQR: 12–17) children (*p* < 0.01).

**Table 2:** Propensity score weighted hazard ratios (95% CI) of emotional outcomes in children according to maternal exposure to SSRI (N06AB) in utero (1997–2015)

OUTCOME	Exposure	Person Yrs	Events	HR (95% CI)	
**Any diagnosis or medication**	No	8,699,120	33,367	1.00 (ref.)	
	Yes	130,074	856	**1.55 (1.44–1.67)**	
**Diagnoses**					
Any depressive disorder^1^	No	8,804,271	5,980	1.00 (ref.)	
	Yes	132,774	121	**1.55 (1.28–1.87)**	
Any anxiety disorder ^2^	No	8,789,611	9,466	1.00 (ref.)	
	Yes	132,421	225	**1.57 (1.36–1.80)**	
Adjustment disorder ^3^	No	8,756,212	18,555	1.00 (ref.)	
	Yes	131,662	438	**1.28 (1.16–1.42)**	
Any diagnosis	No	8,711,814	29,831	1.00 (ref.)	
	Yes	130,377	771	**1.50 (1.39–1.62)**	
**Medications**					
Any SSRI^4^	No	8,797,801	8,202	1.00 (ref.)	
	Yes	132,506	217	**2.18 (1.89–2.52)**	
Any TCA^5^	No	8,816,506	1,341	1.00 (ref.)	
	Yes	133,111	22	1.22 (0.78–1.90)	
Other antidepressants ^6^	No	8,818,418	1,662	1.00 (ref.)	
	Yes	133,097	33	**1.67 (1.16–2.39)**	
Any medication	No	8,791,357	10,116	1.00 (ref.)	
	Yes	132,422	245	**1.96 (1.71–2.24)**	

^1^ ICD10 F32-39; ^2^ ICD10 F40-42; ^3^ ICD10 F43-49; ^4^ ICD10 F32-49; ^5^ATC N06AB; ^6^ ATC N06AA; ^7^ATC N06AX + N06AF + N06AG

HR = Propensity score weighted hazard ratio; **Ref**: Reference (unexposed) group. TCA: tricyclic antidepressant

Significant associations are bolded

The above associations were similar when using PS-matching instead of weighting (**Table S4**), were present in both female (*HR* = 1.46 [1.32–1.62]) and male (*HR* = 1.67 [1.50–1.87]) children (**Table S5)**, and significant across first (*HR* = 1.64 [1.52–1.78]), second (*HR* = 1.64 [1.56–1.93]) and third (*HR* = 1.61 [1.44–1.79]) trimesters of exposures (**Table S6).**

Associations between SSRI exposure and children emotional disorder diagnoses and antidepressant prescriptions were also evident when we restricted our analyses to the 19,875 women who had a documented psychiatric diagnosis during or one year prior to pregnancy (*HR* = 1.38 [1.17–1.63], Table 3). However, the effect size was significantly attenuated when the unexposed group was restricted to women who were previously prescribed, but discontinued SSRI use two years to three months prior to start of pregnancy (*HR* = 1.23 [1.13–1.34] (Table 4) consistent with confounding by indication.

**Table 3:** Propensity score weighted hazard ratios (95% CI) of emotional outcomes in children according to maternal exposure to SSRI (N06AB) in women with a psychiatric diagnosis within one year prior to pregnancy (ICD10 F00-F99) (1997–2015)

OUTCOME	Exposure	Person Yrs	Events	HR (95% CI)	
**Any diagnosis or medication**	No	91,489	505	1.00 (ref.)	
	Yes	47,831	318	**1.38 (1.17–1.63)**	
**Diagnoses**					
Any depressive disorder^1^	No	93,125	84	1.00 (ref.)	
	Yes	48,825	48	**1.59 (1.04–2.42)**	
Any anxiety disorder ^2^	No	92,939	129	1.00 (ref.)	
	Yes	48,661	77	1.25 (0.89–1.75)	
Adjustment disorder ^3^	No	92,298	296	1.00 (ref.)	
	Yes	48,367	181	**1.37 (1.11–1.68)**	
Any diagnosis	No	91,662	458	1.00 (ref.)	
	Yes	47,904	295	**1.36 (1.14–1.61)**	
**Medications**					
Any SSRI^4^	No	93,058	105	1.00 (ref.)	
	Yes	48,756	65	**2.10 (1.48–2.99)**	
Any TCA^5^	No	93,287	10	1.00 (ref.)	
	Yes	48,920	9	2.20 (0.86–5.65)	
Other antidepressants ^6^	No	93,294	26	1.00 (ref.)	
	Yes	48,929	5	0.89 (0.31–2.56)	
Any medication	No	92,978	131	1.00 (ref.)	
	Yes	48,728	73	**1.86 (1.35–2.56)**	

^1^ ICD10 F32-39; ^2^ ICD10 F40-42; ^3^ ICD10 F43-49; ^4^ ICD10 F32-49; ^5^ATC N06AB; ^6^ ATC N06AA; ^7^ATC N06AX + N06AF + N06AG

**HR** = Propensity score weighted hazard ratio; **Ref**: Reference (unexposed) group. **TCA**: tricyclic antidepressant

Significant associations are bolded

**Table 4:** Propensity score weighted hazard ratios (95% CI) of emotional outcomes in children according to maternal exposure to SSRI (N06AB) compared to discontinued use (1997–2015)

OUTCOME	Exposure	Person Yrs	Events	HR (95% CI)	
**Any diagnosis or medication**	No	251,070	1,404	1.00 (ref.)	
	Yes	171,781	1,107	**1.23 (1.13–1.34**)	
**Diagnoses**					
Any depressive disorder^1^	No	255,495	217	1.00 (ref.)	
	Yes	175,331	155	1.22 (0.98–1.51)	
Any anxiety disorder ^2^	No	254,898	365	1.00 (ref.)	
	Yes	174,841	283	1.21 (1.03–1.42)	
Adjustment disorder ^3^	No	253,529	785	1.00 (ref.)	
	Yes	173,837	584	**1.14 (1.02–1.27)**	
Any diagnosis	No	251,540	1,271	1.00 (ref.)	
	Yes	172,183	1,001	**1.21 (1.11–1.31)**	
**Medications**					
Any SSRI^4^	No	255,076	329	1.00 (ref.)	
	Yes	174,989	265	**1.49 (1.26–1.76)**	
Any TCA^5^	No	255,990	45	1.00 (ref.)	
	Yes	175,711	28	1.04 (0.64–1.69)	
Other antidepressants ^6^	No	255,993	67	1.00 (ref.)	
	Yes	175,713	35	1.06 (0.70–1.61)	
Any medication	No	254,891	397	1.00 (ref.)	
	Yes	174,863	300	**1.39 (1.19–1.62)**	

^1^ ICD10 F32-39; ^2^ ICD10 F40-42; ^3^ ICD10 F43-49; ^4^ ICD10 F32-49; ^5^ATC N06AB; ^6^ ATC N06AA; ^7^ATC N06AX + N06AF + N06AG

**HR** = Propensity score weighted hazard ratio; **Ref**: Reference (unexposed) group. **TCA**: tricyclic antidepressant

Significant associations are bolded

### Sensitivity analyses

Associations between SSRI exposure and child emotional disorder diagnoses and antidepressant prescriptions were evident when we [1] excluded women prescribed mood stabilizing or antipsychotic treatment during pregnancy from both groups (*HR* = 1.54 [1.43–1.66], **Table S7**); [4] required at least one SSRI refill during the index pregnancy, as a marker of medication adherence (*HR* = 1.56 [1.43, 1.71], **Table S8**), and when we conducted a complete case analysis restricted to only mother-child dyads with no missing data (*HR* = 1.56 [1.43, 1.71], **Table S9**).

### Specificity to maternal prenatal exposure

To explore specificity and the extent to which the aforementioned associations were likely pharmacologically driven, we used paternal SSRI use during the index pregnancy and maternal SSRI use only after the index pregnancy as additional comparison groups. After adjusting for paternal depression using paternal PS-weighting, paternal SSRI exposure during the corresponding pregnancy was also associated with child emotional disorders (*HR* = 1.48 [1.37–1.60], **Table S10**). Importantly, this association remained when excluding mothers with psychiatric diagnoses or antidepressant prescriptions (*HR* = 1.46 [1.34–1.58], right columns), suggesting that paternal use did not solely serve as a proxy for maternal use. Similarly, after controlling for maternal postnatal depression and maternal prenatal PS-weighting, maternal SSRI postnatal use within two years postpartum was associated with higher rates of child psychiatric outcomes (*HR* = 1.42 [1.35–1.49], **Table S11**).

Finally, we ran a combined model including maternal pre- and post-natal SSRI use and paternal prenatal SSRI use. As shown in **Table S12**, maternal SSRI use [1] only during pregnancy (adjusted HR:1.34 [1.26–1.42]); [2] only after pregnancy (HR: 1.48 [1.41–1.55]); and [3] both during and after pregnancy (HR = 1.58 [1.48–1.69]) were associated with increased emotional disorder diagnoses and antidepressant prescriptions in the children. Paternal use also continued to have a significant, albeit smaller, association with child outcomes (HR = 1.19 [1.11–1.28]).

**Fig. 3:**
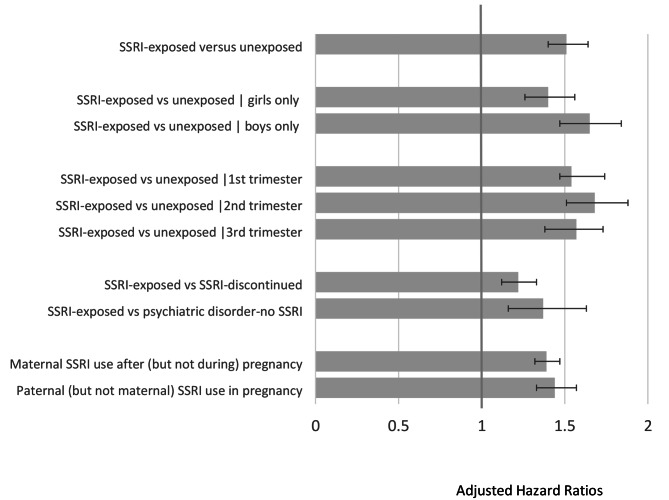
Summary of findings. This chart summarizes the results for our primary outcomes (namely, the first diagnosis of any depressive, anxiety or adjustment disorder, or an antidepressant medication redemption (the individual diagnostic and medication components comprising the outcomes are detailed in the respective tables). All hazard ratios (HRs) are propensity score weighted; a HR > 1 indicates that SSRI exposure is associated with greater risk for the outcome; <1 would indicate lower risk. We find that overall, SSRI exposure overall is associated with a 1.5 fold increased risk for our primary psychiatric outcome (top, details in Table 2); [2] that this association is found in both female and male children (Table S5); [3] that it is observed in each trimester of use (Table S10) and [4] that the risk of the primary outcome is greater in children who were exposed to SSRIs in utero than those who whose mothers had previously used an SSRI but discontinued it prior to pregnancy (Table S7), and to those whose mothers had a documented psychiatric disorder during pregnancy but no medication (Table S6). Finally, we found that maternal SSRI use only after (but no during) pregnancy (Table S12), and paternal SSRI use during a corresponding pregnancy when the mother did not use SSRI (Table S11) were also associated with increased risk in the children. These findings collectively lead us to conclude that whereas SSRI exposure is associated with worse outcomes related to depressive and anxiety disorders and their treatment, in the children, these are not likely to be driven primarily by the medication, but rather by parental illness severity or other confounders

The main findings of this manuscript are visually summarized in Fig. 3.

## Discussion

The increased risk for depression and poor functioning in the children of depressed parents is among the best replicated findings in psychiatry [31]. In this study, we tested whether prenatal SSRI antidepressant exposure further increased the risk to the children. This was based on hypotheses generated from animal studies showing that changes to serotonin levels during gestation could alter the development of limbic neurocircuitry and increase the risk for adolescent depressive and anxiety-like phenotypes [8, 32–34]

We found that children with *in utero* exposure to SSRIs had both higher and earlier rates of emotional disorders and antidepressant medication prescriptions, than unexposed children or children gestationally exposed to maternal psychiatric illness but no medication. These findings comport with the aforementioned preclinical studies [13, 35] and some prior human studies showing increased psychopathology in antidepressant-exposed children [17–19]. At the same time, we found that when we compared children exposed to SSRIs to those whose mothers discontinued SSRI medications prior to conception, the effect size was significantly attenuated (HR from 1.55 to 1.23). Moreover, maternal SSRI use only after pregnancy and paternal SSRI use during the corresponding pregnancy–even in the absence of any prenatal SSRI exposure – were each similarly associated with adverse child outcomes. These findings together suggest that the adverse outcomes described in this study likely result from a combination of confounding by indication or severity of illness and other environmental or genetic factors and are unlikely to be primarily attributable to gestational exposure.

Recognizing the burden of untreated illness in pregnancy, the US task force for prevention of depression now recommends depression screening for all women of child-bearing age. For pregnant women with milder depressive symptoms, discontinuation or switch to evidence-based psychotherapy is often recommended [36–38]. Outside of pregnancy, several studies have demonstrated that successfully treating a mother’s depression improves her child’s symptoms and functioning as well [39–41]. Although SSRI treatment of mothers during pregnancy might also be expected to improve child outcomes, to the contrary, we find that the children of mothers treated with SSRIs during pregnancy have increased rates of emotional disorders and antidepressant use. This study does not resolve the question of whether SSRI use in the peripartum period is beneficial or detrimental to child development. However, maternal SSRI use during or after pregnancy clearly predict smore adverse outcomes, and exposure – regardless of the mechanism – may index a higher risk group of children who could be targeted with screening or preventive interventions. Such measures could prevent or delay emotional disorders in these children, reducing the climbing rates of depression and anxiety in adolescence,[42] and the more severe course associated with earlier onset [43]. [Similar findings and conclusions were reached in a recent US claims data study on earlier neurodevelopmental disorders including autism and attention deficit/hyperactivity disorders [44]] Such preventative efforts may be particularly pertinent to boys, as we note that even though the overall prevalence of depressive and anxiety disorders was higher in female children, the SSRI-attributed increase in rates was greater among males. This suggests that the SSRI exposure could be ‘leveling out’ expected sex differences in adolescent susceptibility to depression and anxiety. We did not find formal sex interactions, however, so future work will need to confirm these patterns and further determine whether there is a biological basis for any such differential susceptibility.

### Strengths & limitations

Though previous studies have examined the risk of prenatal antidepressant exposures on child emotional development, our findings expand the literature in several ways. Using Danish National registers encompassing all children and their parents in an unselected nationwide population with very long follow-up (up to 22 years) is a major strength that minimizes most selection biases. We also included multiple diagnostic and medication proxies of our primary outcomes to maximize our coverage of the risk to the children. We used multiple propensity score approaches to account for potential confounding and, for the first time, nested data from the Danish National Birth Cohort to examine the impact of subclinical factors, which are typically not quantifiable from register data. What we found through this approach, however, is that though ameliorated, not all differences are fully eliminated, and residual confounding likely remains. This is particularly true for self-reported psychopathology from the DNBC. Given that we are finding such constraints in data from the Nordic countries – where there are generally fewer biases and barriers to healthcare access – more robust methodologies will be needed to generate maximally comparable groups when using data from countries or healthcare settings where there may be larger confounding gradients (e.g., socioeconomic) driving medication use. At the same time, birth cohorts that can more directly and granularly assess the pregnancy and postpartum period may help to quantify the components of risk to the offspring generation predisposed to by the medications versus maternal illness or third factors.

Other limitations – shared by many register studies – include the reliance on diagnoses obtained from hospital clinics (excluding individuals treated by general practitioners), dispensations of prescriptions typically in 3-monthly packs, precluding our ability to test for further temporal specificity. As this is an observational study, misclassification, including differential misclassification by either exposure or outcome cannot be fully ruled out. Finally, while the study population was unselected and findings should thus generalize to the Danish population, they may not generalize to other countries with different racial and ethnic makeup, or different cultures around antidepressant use or prescribing practices.

## Conclusion

We extend previous findings that children exposed to maternal SSRI use during pregnancy are themselves at increased risk for internalizing (depressive, anxiety, adjustment) disorders till age 22, but show that these increases may not be exclusively driven by pharmacological effects. This does not indicate that SSRIs do not confer direct risks; indeed, evidence from animal [13, 14, 45] and some clinical [46, 47] studies demonstrate alterations in infant brain structure and connectivity following exposure to SSRI or other serotonin-altering agents. Rather, they suggest that the associations with clinical disorders may be more strongly driven by parental depression and its correlates. Regardless of mechanism, the SSRI-exposed children reflect a higher-risk group for depression and anxiety than the general population and may warrant increased clinical screening as they pass through the age of risk.

## Electronic supplementary material

Below is the link to the electronic supplementary material.

Supplementary Material 1

## Data Availability

The data were obtained from Danish National Patient Register, the Danish Medical Birth Register, the Danish Prescription Register, the Danish Civil Register, and the Danish National Birth Cohort.
