# Supplementary material for: Prenatal antidepressant exposure and emotional disorders until age 22: a danish register study
Source: Child Adolesc Psychiatry Ment Health. 2023 Jun 16;17:73. doi: 10.1186/s13034-023-00624-9 (PMC10276495; doi:10.1186/s13034-023-00624-9)
Supplement: Supplementary file 1 — Supplementary Material 1 [file 13034_2023_624_MOESM1_ESM.docx]

Prenatal exposure to selective serotonin reuptake inhibitors (SSRIs) and risk of emotional disorders and antidepressant use in the children until age 22: a Danish register study.

**Supplementary Materials**

Contains 12 tables (Tables S1-12) and one figure (Figure S1).

**Table S1**: Outcome definition by International Classification of Diseases codes, version 10 (ICD) and Anatomical Therapeutic Chemical Classification (ATC) codes

| **Outcome definitions** |  |  |  |  |
| --- | --- | --- | --- | --- |
|  |  |  |  |  |
| **Diagnoses** |  |  |  |  |
| Depressive disorders | ICD10 | F32-39* |  |  |
| Anxiety disorders | ICD10 | F40-42* |  |  |
| Adjustment disorders | ICD10 | F43-49* |  |  |
| Emotional disorders with onset specific to childhood | ICD10 | F93* |  |  |
|  |  |  |  |  |
| **Prescriptions** |  |  |  |  |
| SSRI | ATC | N06AB* |  |  |
| TCA | ATC | N06AA* |  |  |
| Other antidepressants | ATC | N06AX*, N06AF*, N06AG* |  |  |
| * Including subgroups. | | |  |  |
|  |  |  |  |  |
|  |  |  |  |  |

**Table S2**: Characteristics of the study cohort according to SSRI exposure during pregnancy (1997-2015) with full description of all variables included in the propensity score weighing and information on missing numbers.

|  | **Unweighted** |  |  |  | **Weighted** |  |  |
| --- | --- | --- | --- | --- | --- | --- | --- |
|  | **Exposed** | **Unexposed** | **SMD1** |  | **Exposed** | **Unexposed** | **SMD2** |
|  | (n=15,651) | (n=896,818) |  |  | (n=15,651) | (n=14,959) |  |
| **MOTHERS** | N (%) | N (%) |  |  | N (%) | N (%) |  |
| **Age** |  |  |  |  |  |  |  |
| Median (*IQR, years)* | 30 (27-34) | 30 (27-33) | . |  | 30 (27-34) | 30 (26-34) | 0.02 |
| <20 years | 266 (1.7) | 12,063 (1.3) | 0.03 |  | 266 (1.7) | 292 (2.0) | 0.02 |
| 20-24 years | 2089 (13) | 99,083 (11) | 0.07 |  | 2,089 (13.3) | 1,967 (13.2) | 0.01 |
| 25-29 years | 4648 (30) | 294,163 (33) | 0.07 |  | 4,648 (29.7) | 4,593 (30.7) | 0.02 |
| 30-34 years | 5319 (34) | 321,433 (36) | 0.04 |  | 5,319 (34.0) | 4,975 (33.3) | 0.02 |
| 35-39 years | 2764 (18) | 144,011 (16) | 0.04 |  | 2,764 (17.7) | 2,562 (17.1) | 0.01 |
| 40+ years | 565 (3.6) | 26,065 (2.9) | 0.04 |  | 565 (3.6) | 571 (3.8) | 0.01 |
| **Parity** |  |  |  |  |  |  |  |
| First childbirth | 6742 (43) | 388,114 (43) | 0.00 |  | 6,742 (43.1) | 6,390 (42.7) | 0.01 |
| 2-3 childbirth | 7770 (50) | 460,634 (52) | 0.03 |  | 7,770 (49.6) | 7,536 (50.4) | 0.01 |
| 4+ childbirth | 1084 (7.0) | 45,665 (5.1) | 0.08 |  | 1,084 (6.9) | 984 (6.6) | 0.01 |
| **Body mass index** |  |  |  |  |  |  |  |
| Underweight | 560 (4.6) | 25,091 (4.3) | 0.04 |  | 560 (3.6) | 505 (3.4) | 0.01 |
| Normal weight | 6689 (55) | 358,529 (62) | 0.06 |  | 6,689 (42.7) | 6,447 (43.1) | 0.01 |
| Overweight | 2748 (23) | 122,910 (21) | 0.11 |  | 2,748 (17.6) | 2,596 (17.4) | 0.01 |
| Obese, class 1 | 1282 (11) | 46,557 (8.0) | 0.12 |  | 1,282 (8.2) | 1,185 (7.9) | 0.01 |
| Obese, class 2 & 3 | 866 (7.1) | 27,011 (4.7) | 0.12 |  | 866 (5.5) | 788 (5.3) | 0.01 |
| **Smoking in pregnancy** |  |  |  |  |  |  |  |
| No smoking | 10,897 (73) | 706,880 (84) | 0.21 |  | 10,897 (69.6) | 10,656 (71.2) | 0.04 |
| Light (1-10 cigs/day) | 2775 (19) | 103,016 (12) | 0.18 |  | 2,775 (17.7) | 2,541 (17.0) | 0.02 |
| Heavy (11+ cigs/day) | 1259 (8.4) | 34,332 (4.1) | 0.18 |  | 1,259 (8.0) | 1,079 (7.2) | 0.03 |
| **Marital status** |  |  |  |  |  |  |  |
| Unmarried | 8616 (55) | 426,635 (48) | 0.15 |  | 8,616 (55.1) | 8,055 (53.8) | 0.02 |
| Married/registered  partnership | 7034 (45) | 470,065 (52) | 0.15 |  | 7,034 (44.9) | 6,904 (46.2) | 0.02 |
| **Employment status** |  |  |  |  |  |  |  |
| Unemployed | 6503 (42) | 246,077 (27) | 0.30 |  | 6,503 (41.6) | 5,812 (38.9) | 0.06 |
| Student | 940 (6.0) | 53,299 (5.9) | 0.00 |  | 940 (6.0) | 925 (6.2) | 0.01 |
| Employed | 7935 (51) | 577,870 (64) | 0.28 |  | 7,935 (50.7) | 7,947 (53.1) | 0.05 |
| Self-employed | 272 (1.7) | 19,513 (2.2) | 0.03 |  | 272 (1.7) | 275 (1.8) | 0.01 |
| **Highest education** |  |  |  |  |  |  |  |
| Short (7-10 years) | 4428 (29) | 164,174 (19) | 0.24 |  | 4,428 (28.3) | 3,919 (26.2) | 0.05 |
| Vocational training | 4357 (28) | 261,434 (30) | 0.03 |  | 4,357 (27.8) | 4,292 (28.7) | 0.02 |
| Medium (11-13 years) | 2266 (15) | 139,282 (16) | 0.03 |  | 2,266 (14.5) | 2,181 (14.6) | 0.00 |
| Long (13+ years) | 4279 (28) | 309,088 (35) | 0.15 |  | 4,279 (27.3) | 4,274 (28.6) | 0.03 |
| **Income** |  |  |  |  |  |  |  |
| First quartile (Lowest) | 5318 (34) | 203,342 (23) | 0.25 |  | 5,318 (34.0) | 4,706 (31.5) | 0.05 |
| Second quartile | 6227 (40) | 342,024 (38) | 0.03 |  | 6,227 (39.8) | 6,143 (41.1) | 0.03 |
| Third quartile | 2934 (19) | 235,792 (26) | 0.18 |  | 2,934 (18.7) | 2,931 (19.6) | 0.02 |
| Forth quartile (Highest) | 1159 (7.4) | 114,734 (13) | 0.18 |  | 1,159 (7.4) | 1,168 (7.8) | 0.02 |
| **CHILDREN** |  |  |  |  |  |  |  |
| **Gender** |  |  |  |  |  |  |  |
| Male | 8098 (52) | 460,255 (51) | 0.01 |  | 8,098 (51.7) | 7,676 (51.3) | 0.01 |
| Female | 7553 (48) | 436,563 (49) | 0.01 |  | 7,553 (48.3) | 7,283 (48.7) | 0.01 |
| **Apgar score (5 min)** |  |  |  |  |  |  |  |
| < 7 | 243 (1.6) | 6603 (0.7) | 0.08 |  | 243 (1.6) | 120 (0.8) | 0.07 |
| ≥7+ | 15,282 (98) | 883,940 (99) | 0.07 |  | 15,282 (97.6) | 14,741 (98.5) | 0.07 |
| **Birth year** |  |  |  |  |  |  |  |
| 1997-2003 | 3017 (19) | 291,736 (33) | 0.31 |  | 3,017 (19.3) | 2,966 (19.8) | 0.01 |
| 2004-2009 | 6218 (40) | 322,830 (36) | 0.08 |  | 6,218 (39.7) | 6,402 (42.8) | 0.06 |
| 2010-2015 | 6416 (41) | 282,252 (31) | 0.20 |  | 6,416 (41.0) | 5,591 (37.4) | 0.07 |
| **VARIABLES USED IN PROPENSITY SCORES** | | | | | | | |
| **Visits general practitioner*** |  |  |  |  |  |  |  |
| 0 | 124 (0.8) | 18,380 (2.0) | 0.11 |  | 124 (0.8) | 113 (0.8) | 0.00 |
| 1-2 | 288 (1.8) | 39,517 (4.4) | 0.15 |  | 288 (1.8) | 265 (1.8) | 0.00 |
| 3+ | 15,239 (97) | 838,921 (94) | 0.18 |  | 15,239 (97.4) | 14,581 (97.5) | 0.01 |
| **Outpatient visits*** |  |  |  |  |  |  |  |
| 0 | 9450 (60) | 599,326 (67) | 0.13 |  | 9,450 (60.4) | 9,223 (61.7) | 0.03 |
| 1-2 | 4702 (30) | 240,575 (27) | 0.07 |  | 4,702 (30.0) | 4,407 (29.5) | 0.01 |
| 3+ | 1499 (9.6) | 56,917 (6.3) | 0.12 |  | 1,499 (9.6) | 1,329 (8.9) | 0.02 |
| **Hospital admissions*** |  |  |  |  |  |  |  |
| 0 | 12,281 (78) | 718,036 (80) | 0.04 |  | 12,281 (78.5) | 11,747 (78.5) | 0.00 |
| 1-2 | 3004 (19) | 165,092 (18) | 0.02 |  | 3,004 (19.2) | 2,878 (19.2) | 0.00 |
| 3+ | 366 (2.3) | 13,690 (1.5) | 0.06 |  | 366 (2.3) | 335 (2.2) | 0.01 |
| **Filled prescriptions,**  **non-psychiatric**** |  |  |  |  |  |  |  |
|  | 3803 (24) | 275,573 (31) | 0.14 |  | 3,803 (24.3) | 3,691 (24.7) | 0.01 |
|  | 6977 (45) | 411,929 (46) | 0.03 |  | 6,977 (44.6) | 6,767 (45.2) | 0.01 |
|  | 4871 (31) | 209,316 (23) | 0.18 |  | 4,871 (31.1) | 4,501 (30.1) | 0.02 |
|  | 3803 (24) | 275,573 (31) | 0.14 |  | 3,803 (24.3) | 3,691 (24.7) | 0.01 |
| **Visits private psychiatrists*** |  |  |  |  |  |  |  |
|  | 14,674 (94) | 892,161 (99) | 0.32 |  | 14,674 (93.8) | 14,596 (97.6) | 0.19 |
|  | 977 (6.2) | 4657 (0.5) | 0.32 |  | 977 (6.2) | 363 (2.4) | 0.19 |
| **Outpatient visits*** |  |  |  |  |  |  |  |
| 0 | 15,181 (97) | 894,197 (99.7) | 0.21 |  | 15,181 (97.0) | 14,648 (97.9) | 0.06 |
| 1-2 | 462 (3.0) | 2582 (0.3) | 0.21 |  | 462 (3.0) | 307 (2.0) | 0.06 |
| 3+ | 8 (0.1) | 39 (0.0) | 0.03 |  | 8 (0.1) | 5 (0.0) | 0.01 |
| **Hospital admissions, psychiatry*** |  |  |  |  |  |  |  |
| 0 | 15,552 (99) | 896,250 (100) | 0.10 |  | 15,552 (99.4) | 14,904 (99.6) | 0.04 |
| 1-2 | 96 (0.6) | 529 (0.1) | 0.10 |  | 96 (0.6) | 52 (0.3) | 0.04 |
| 3+ | (n<5) | 39 (0.0) | 0.01 |  | <5 | <5 | 0.00 |
| **Filled prescriptions, psychiatric*** |  |  |  |  |  |  |  |
| No | 14,553 (93) | 886,495 (99) | 0.30 |  | 14,553 (93.0) | 14,414 (96.4) | 0.15 |
| Yes | 1098 (7.0) | 10,323 (1.2) | 0.30 |  | 1,098 (7.0) | 545 (3.6) | 0.15 |
| **History of psychiatric diagnosis *** |  |  |  |  |  |  |  |
| No | 7588 (48) | 779,440 (87) | 0.90 |  | 7,588 (48.5) | 7,515 (50.2) | 0.04 |
| Yes | 8063 (52) | 117,378 (13) | 0.90 |  | 8,063 (51.5) | 7,445 (49.8) | 0.04 |
| **History of suicide attempts** |  |  |  |  |  |  |  |
| No | 15,317 (98) | 892,694 (100) | 0.15 |  | 15,317 (97.9) | 14,716 (98.4) | 0.04 |
| Yes | 334 (2.1) | 4124 (0.5) | 0.15 |  | 334 (2.1) | 243 (1.6) | 0.04 |
| **Filled prescriptions, other antidepressants**** |  |  |  |  |  |  |  |
| No | 14,986 (96) | 894,114 (100) | 0.27 |  | 14,986 (95.8) | 14,777 (98.8) | 0.19 |
| Yes | 665 (4.2) | 2704 (0.3) | 0.27 |  | 665 (4.2) | 183 (1.2) | 0.19 |
| **FREQUENCY OF MISSING INFORMATION** | | | | | | | |
| Age group | 0 | 0 | . |  | 0 | 0 | . |
| Parity | 55 (0.4) | 2405 (0.3) | 0.01 |  | 55 (0.4) | 48 (0.3) | 0.00 |
| BMI | 3506 (22) | 316,720 (35) | 0.29 |  | 3,506 (22.4) | 3,438 (23.0) | 0.01 |
| Smoking status | 720 (4.6) | 52,590 (5.9) | 0.06 |  | 720 (4.6) | 683 (4.6) | 0.00 |
| Civil status | (n<5) | 118 (0.0) | 0.01 |  | <5 | 1 (0.0) | 0.00 |
| Socioeconomic status | (n<5) | 59 (0.0) | 0.00 |  | <5 | 0 (0.0) | 0.01 |
| Education level | 321 (2.1) | 22,840 (2.5) | 0.03 |  | 321 (2.1) | 293 (2.0) | 0.01 |
| Annual income | 13 (0.1) | 926 (0.1) | 0.01 |  | 13 (0.1) | 13 (0.1) | 0.00 |
| Apgar score at 5 minutes | 126 (0.8) | 6275 (0.7) | 0.01 |  | 126 (0.8) | 98 (0.7) | 0.02 |
|  |  |  |  |  |  |  |  |
|  |  |  |  |  |  |  |  |
|  |  |  |  |  |  |  |  |

* 1 Year prior to pregnancy

**180 days prior to pregnancy

SMD1: Standard mean difference, prior to propensity score weighting (unadjusted)

SMD2: Standard mean difference, after propensity score weighting (adjusted)

**Table S3**: Characteristics of the DNBC sub cohort on mental health indicators according to SSRI exposure during pregnancy (1997-2003) propensity score weighted according to main propensity score model using register-based information

|  |  |  |  |  |  |  |  |
| --- | --- | --- | --- | --- | --- | --- | --- |
|  | **Unweighted** |  |  |  | **Weighted** |  |  |
| All | **Exposed** | **Unexposed** | **SMD1** |  | **Exposed** | **Unexposed** | **SMD2** |
|  | N =406 | N=76,042 |  |  | N=406 | N= 404 |  |
| **Age** |  |  |  |  |  |  |  |
| Median (IQR, years) | 30 (27-33) | 30 (27-33) |  |  | 30 (27-33) | 30 (27-33) | 0.05 |
|  |  |  |  |  |  |  |  |
| **Parity** |  |  |  |  |  |  |  |
| First childbirth | 197 (49) | 35,277 (47) | 0.04 |  | 197 (49) | 190 (47) | 0.03 |
| >1 childbirth | 208 (51) | 40,417 (53) | 0.04 |  | 208 (51) | 211 (52) | 0.02 |
| **How would you characterize your health in general?** |  |  |  |  |  |  |  |
| Very good | 134 (33) | 39,906 (52) | 0.40 |  | 134 (33) | 190 (47) | 0.29 |
| Average | 228 (56) | 33,313 (44) | 0.25 |  | 228 (56) | 191 (47) | 0.18 |
| Not so good | 44 (11) | 2790 (3.7) | 0.28 |  | 44 (11) | 23 (5.6) | 0.19 |
| **Do you worry about the coming birth?** |  |  |  |  |  |  |  |
| Not at all | 136 (33) | 31,599 (42) | 0.17 |  | 136 (33) | 159 (39) | 0.12 |
| A little | 207 (51) | 37,534 (49) | 0.03 |  | 207 (51) | 197 (49) | 0.04 |
| Very much | 63 (16) | 6841 (9.0) | 0.20 |  | 63 (16) | 47 (12) | 0.11 |
| **Do you worry about your unborn child's health?** |  |  |  |  |  |  |  |
| Not at all | 54 (13) | 12,029 (16) | 0.07 |  | 54 (13) | 63 (16) | 0.06 |
| A little | 215 (53) | 44,530 (59) | 0.11 |  | 215 (53) | 225 (56) | 0.05 |
| Very much | 137 (34) | 19,409 (26) | 0.18 |  | 137 (34) | 116 (29) | 0.11 |
| **Planned pregnancy** |  |  |  |  |  |  |  |
| Yes | 303 (75) | 67,368 (89) | 0.37 |  | 303 (75) | 344 (85) | 0.26 |
| No | 103 (25) | 8651 (11) | 0.37 |  | 103 (25) | 60 (15) | 0.26 |
| **Have you ever suffered from mental disorders or neurosis?** |  |  |  |  |  |  |  |
| No | 90 (22) | 71,074 (93) | 2.09 |  | 90 (22) | 351 (87) | 1.71 |
| Yes | 316 (78) | 4908 (6.5) | 2.09 |  | 316 (78) | 52 (13) | 1.72 |
| **Have you ever suffered from bulimia?** |  |  |  |  |  |  |  |
| No | 356 (88) | 73,952 (97) | 0.37 |  | 356 (88) | 386 (96) | 0.29 |
| Yes | 50 (12) | 2063 (2.7) | 0.37 |  | 50 (12) | 17 (4.2) | 0.30 |
| **Have you ever suffered from anorexia?** |  |  |  |  |  |  |  |
| No | 365 (90) | 74,006 (97) | 0.31 |  | 365 (90) | 386 (96) | 0.22 |
| Yes | 41 (10) | 1973 (2.6) | 0.31 |  | 41 (10) | 17 (4.3) | 0.23 |
| **Alcohol consumption before pregnancy (glass/week)** |  |  |  |  |  |  |  |
| 0 | 66 (16) | 10,006 (13) | 0.09 |  | 66 (16) | 66 (16) | 0.00 |
| 1-3 | 188 (47) | 40,468 (53) | 0.14 |  | 188 (46) | 206 (51) | 0.10 |
| 4-6 | 75 (19) | 15,334 (20) | 0.04 |  | 75 (18) | 74 (18) | 0.00 |
| 7+ | 75 (19) | 9882 (13) | 0.15 |  | 75 (18) | 55 (14) | 0.13 |
| **Alcohol consumption in pregnancy (first trimester)** |  |  |  |  |  |  |  |
| 0 | 244 (60) | 42,152 (55) | 0.09 |  | 244 (60) | 237 (59) | 0.03 |
| 1-3 | 147 (36) | 32,047 (42) | 0.12 |  | 147 (36) | 156 (39) | 0.05 |
| 4-6 | 12 (3.0) | 1537 (2.0) | 0.06 |  | 12 (3.0) | 9 (2.2) | 0.05 |
| 7+ | (n<5) | 222 (0.3) | 0.03 |  | <5 | <5 | 0.02 |
| **Self-reported smoking in pregnancy**  **(first trimester)** |  |  |  |  |  |  |  |
| Not smoking | 217 (53) | 56,210 (74) | 0.44 |  | 217 (53) | 255 (63) | 0.20 |
| Smoking cessation in pregnancy | 56 (14) | 7217 (9.5) | 0.13 |  | 56 (14) | 43 (11) | 0.09 |
| Smoking | 133 (33) | 12,613 (17) | 0.38 |  | 133 (33) | 105 (26) | 0.15 |
| 1-3 | 90 (22) | 71,074 (93) | 2.09 |  | 90 (22) | 351 (87) | 1.71 |
| 4-6 | 316 (78) | 4908 (6.5) | 2.09 |  | 316 (78) | 52 (13) | 1.72 |
| 7+ |  |  |  |  |  |  |  |
| **Missing data / did not want to answer** |  |  |  |  |  |  |  |
| Age | - | - |  |  | - | - |  |
| Parity | (n<5) | 348 (0.5) | 0.04 |  | <5 | <5 | 0.07 |
| Health in general | - | 32 (0.0) | 0.03 |  | - | 0 (0.1) | 0.04 |
| Coming birth | - | 68 (0.1) | 0.04 |  | - | 0 (0.1) | 0.05 |
| Health of unborn child | - | 74 (0.1) | 0.04 |  | - | 0 (0.1) | 0.04 |
| Planned pregnancy | - | 23 (0.0) | 0.02 |  | - | 0 (0.0) | 0.02 |
| Mental disorders | - | 60 (0.1) | 0.04 |  | - | 0 (0.1) | 0.05 |
| Bulimia | - | 27 (0.0) | 0.03 |  | - | 0 (0.1) | 0.03 |
| Anorexia | - | 63 (0.1) | 0.04 |  | - | 0 (0.1) | 0.05 |
| Alcohol consumption before pregnancy | (n<5) | 352 (0.5) | 0.00 |  | <5 | <5 | 0.01 |
| Alcohol consumption in first trimester | (n<5) | 84 (0.1) | 0.03 |  | <5 | 1 (0.1) | 0.02 |
| Smoking in pregnancy | - | (n<5) | 0.01 |  | - | 0 (0.0) | 0.01 |
|  |  |  |  |  |  |  |  |

SMD1: Standard mean difference, prior to propensity score weighting (unadjusted)

SMD2: Standard mean difference, after propensity score weighting (adjusted)

**Table S4:** Propensity score matched hazard ratios (95% CI) of emotional outcomes in children according to maternal exposure to SSRI (N06AB) in utero (1997-2015)

|  |  |  | | |  |
| --- | --- | --- | --- | --- | --- |
| **OUTCOME** | **Exposure** | **Person Yrs** | **Events** | **HR (95% CI)** |  |
| ***Any diagnosis or medication*** | No | 902,071 | 3,901 | 1.00 (ref.) |  |
|  | Yes | 109,660 | 701 | **1.48 (1.37-1.61)** |  |
| ***Diagnoses*** |  |  |  |  |  |
| Any depressive disorder^1^ | No | 914,649 | 573 | 1.00 (ref.) |  |
|  | Yes | 111,874 | 108 | **1.50 (1.22-1.84)** |  |
| Any anxiety disorder ^2^ | No | 912,916 | 1,006 | 1.00 (ref.) |  |
|  | Yes | 111,569 | 193 | **1.55 (1.33-1.81)** |  |
| Adjustment disorder ^3^ | No | 908,287 | 2,337 | 1.00 (ref.) |  |
|  | Yes | 111,009 | 347 | **1.21 (1.08-1.35)** |  |
| Any diagnosis | No | 903,294 | 3,584 | 1.00 (ref.) |  |
|  | Yes | 109,893 | 633 | **1.45 (1.34-1.58)** |  |
| ***Medications*** |  |  |  |  |  |
| Any SSRI^4^ | No | 914,070 | 711 | 1.00 (ref.) |  |
|  | Yes | 111,682 | 184 | **2.07 (1.76-2.44)** |  |
| Any TCA^5^ | No | 915,779 | 113 | 1.00 (ref.) |  |
|  | Yes | 112,167 | 20 | **1.42 (0.89-2.29)** |  |
| Other antidepressants ^6^ | No | 915,875 | 140 | 1.00 (ref.) |  |
|  | Yes | 112,155 | 32 | **1.72 (1.17-2.53)** |  |
| Any medication | No | 913,447 | 888 | 1.00 (ref.) |  |
|  | Yes | 111,602 | 210 | **1.90 (1.63-2.20)** |  |

^1^ ICD10 F32-39; ^2^ ICD10 F40-42; ^3^ ICD10 F43-49; ^4^ ICD10 F32-49; ^5^ATC N06AB; ^6^ ATC N06AA; ^7^ATC N06AX+ N06AF + N06AG

**HR**=Propensity score weighted hazard ratio; **Ref**: Reference (unexposed) group. **TCA**: tricyclic antidepressant.

Significant associations are bolded.

**Table S5**: Propensity score weighted hazard ratios (95% CI) of emotional outcomes in children according to maternal exposure to SSRI (N06AB) in utero stratified by sex (1997-2015)

|  |  |  | **GIRLS** |  |  |  | **BOYS** |  |  |
| --- | --- | --- | --- | --- | --- | --- | --- | --- | --- |
| **OUTCOME** | **Exposure** |  | **Person Yrs** | **Events** | **HR (95% CI)** |  | **Person Yrs** | **Events** | **HR (95% CI)** |
| ***Any diagnosis or medication*** | No |  | 3,974,167 | 17,511 | 1.00 (ref.) |  | 4,268,685 | 13,443 | 1.00 (ref.) |
|  | Yes |  | 61,957 | 422 | **1.46 (1.32-1.62)** |  | 67,151 | 426 | **1.67 (1.50-1.85)** |
| ***Diagnoses*** |  |  |  |  |  |  |  |  |  |
| Any depressive disorder^1^ | No |  | 4,023,496 | 3,648 | 1.00 (ref.) |  | 4,317,400 | 1,790 | 1.00 (ref.) |
|  | Yes |  | 63,188 | 78 | **1.62 (1.28-2.05)** |  | 68,634 | 43 | **1.53 (1.11-2.12)** |
| Any anxiety disorder ^2^ | No |  | 4,018,069 | 5,099 | 1.00 (ref.) |  | 4,309,515 | 3,549 | 1.00 (ref.) |
|  | Yes |  | 63,072 | 110 | **1.34 (1.09-1.64)** |  | 68,367 | 117 | **1.94 (1.59-2.36)** |
| Adjustment disorder ^3^ | No |  | 3,999,337 | 10,208 | 1.00 (ref.) |  | 4,296,516 | 7,034 | 1.00 (ref.) |
|  | Yes |  | 62,709 | 218 | **1.17 (1.02-1.35)** |  | 68,005 | 214 | **1.42 (1.23-1.64)** |
| Any diagnosis | No |  | 3,979,958 | 15,727 | 1.00 (ref.) |  | 4,274,859 | 11,888 | 1.00 (ref.) |
|  | Yes |  | 62,100 | 374 | **1.38 (1.24-1.54)** |  | 67,301 | 392 | **1.65 (1.48-1.84)** |
| ***Medications*** |  |  |  |  |  |  |  |  |  |
| Any SSRI^4^ | No |  | 4,021,043 | 4,658 | 1.00 (ref.) |  | 4,313,582 | 2,869 | 1.00 (ref.) |
|  | Yes |  | 63,070 | 126 | **2.12 (1.76-2.57)** |  | 68,495 | 88 | **2.33 (1.86-2.92)** |
| Any TCA^5^ | No |  | 4,031,596 | 636 | 1.00 (ref.) |  | 4,320,669 | 600 | 1.00 (ref.) |
|  | Yes |  | 63,393 | 15 | **1.85 (1.08-3.19)** |  | 68,762 | 6 | 0.69 (0.30-1.58) |
| Other antidepressants ^6^ | No |  | 4,031,662 | 1,057 | 1.00 (ref.) |  | 4,321,971 | 588 | 1.00 (ref.) |
|  | Yes |  | 63,394 | 22 | **1.86 (1.20-2.89)** |  | 68,746 | 10 | 1.25 (0.64-2.42) |
| Any medication | No |  | 4,018,129 | 5,613 | 1.00 (ref.) |  | 4,310,503 | 3,717 | 1.00 (ref.) |
|  | Yes |  | 63,032 | 144 | **2.02 (1.70-2.41)** |  | 68,451 | 97 | **1.89 (1.53-2.34)** |

^1^ ICD10 F32-39; ^2^ ICD10 F40-42; ^3^ ICD10 F43-49; ^4^ ICD10 F32-49; ^5^ATC N06AB; ^6^ ATC N06AA; ^7^ATC N06AX+ N06AF + N06AG

**HR**=Propensity score weighted hazard ratio; **Ref**: Reference (unexposed) group. **TCA**: tricyclic antidepressant.

Significant associations are bolded.

**Table S6:** Propensity score weighted hazard ratios (95% CI) of emotional outcomes in children according to maternal exposure to SSRI (N06AB) in utero stratified by trimester (1997-2015)

|  |  |  | **First Trimester Exposure** | | |  | **Second Trimester Exposure** | | |  | **Third Trimester Exposure** | | |
| --- | --- | --- | --- | --- | --- | --- | --- | --- | --- | --- | --- | --- | --- |
| **OUTCOME** | **Exposure** |  | **Person Yrs** | **Events** | **HR (95% CI)** |  | **Person Yrs** | **Events** | **HR (95% CI)** |  | **Person Yrs** | **Events** | **HR (95% CI)** |
| ***Any diagnosis or medication*** | No |  | 7,862,330 | 29,482 | 1.00 (ref.) |  | 6,624,341 | 21,779 | 1.00 (ref.) |  | 7,104,670 | 23,764 | 1.00 (ref.) |
|  | Yes |  | 91,546 | 621 | **1.64 (1.52-1.78**) |  | 60,807 | 409 | **1.74 (1.56-1.93)** |  | 61,450 | 391 | **1.61 (1.44-1.79)** |
| ***Diagnoses*** |  |  |  |  |  |  |  |  |  |  |  |  |  |
| Any depressive disorder^1^ | No |  | 7,955,207 | 5,083 | 1.00 (ref.) |  | 6,691,179 | 3,208 | 1.00 (ref.) |  | 7,177,778 | 3,628 | 1.00 (ref.) |
|  | Yes |  | 93,569 | 82 | **1.53 (1.22-1.92)** |  | 62,151 | 49 | **1.66 (1.24-2.23)** |  | 62,673 | 48 | **1.53 (1.13-2.08)** |
| Any anxiety disorder ^2^ | No |  | 7,942,312 | 8,260 | 1.00 (ref.) |  | 6,680,899 | 6,052 | 1.00 (ref.) |  | 7,166,545 | 6,678 | 1.00 (ref.) |
|  | Yes |  | 93,250 | 168 | **1.72 (1.47-2.01)** |  | 61,918 | 108 | **1.78 (1.45-2.17)** |  | 62,454 | 103 | **1.61 (1.31-1.98)** |
| Adjustment disorder ^3^ | No |  | 7,912,160 | 16,498 | 1.00 (ref.) |  | 6,658,461 | 12,508 | 1.00 (ref.) |  | 7,142,534 | 13,533 | 1.00 (ref.) |
|  | Yes |  | 92,748 | 317 | 1.36 (1.21-1.52) |  | 61,626 | 194 | 1.33 (1.14-1.54) |  | 62,206 | 188 | 1.22 (1.05-1.42) |
| Any diagnosis | No |  | 7,873,387 | 26,410 | 1.00 (ref.) |  | 6,630,981 | 19,952 | 1.00 (ref.) |  | 7,112,166 | 21,675 | 1.00 (ref.) |
|  | Yes |  | 91,790 | 560 | **1.60 (1.47-1.74)** |  | 60,965 | 364 | **1.64 (1.47-1.83)** |  | 61,579 | 354 | **1.55 (1.39-1.73)** |
| ***Medications*** |  |  |  |  |  |  |  |  |  |  |  |  |  |
| Any SSRI^4^ | No |  | 7,949,526 | 6,975 | 1.00 (ref.) |  | 6,688,357 | 4,171 | 1.00 (ref.) |  | 7,174,327 | 4,807 | 1.00 (ref.) |
|  | Yes |  | 93,328 | 158 | **2.34 (1.99-2.76)** |  | 61,979 | 99 | **2.87 (2.32-3.54)** |  | 62,528 | 92 | **2.43 (1.94-3.04)** |
| Any TCA^5^ | No |  | 7,965,577 | 1,174 | 1.00 (ref.) |  | 6,696,793 | 816 | 1.00 (ref.) |  | 7,184,447 | 904 | 1.00 (ref.) |
|  | Yes |  | 93,779 | 13 | 1.11 (0.64-1.93) |  | 62,290 | 8 | 0.94 (0.45-1.95) |  | 62,807 | <5 | 0.42 (0.16-1.11) |
| Other antidepressants ^6^ | No |  | 7,967,083 | 1,461 | 1.00 (ref.) |  | 6,698,300 | 675 | 1.00 (ref.) |  | 7,186,056 | 782 | 1.00 (ref.) |
|  | Yes |  | 93,769 | 19 | 1.37 (0.86-2.17) |  | 62,279 | 12 | 2.27 (1.24-4.16) |  | 62,791 | 9 | 1.75 (0.89-3.46) |
| Any medication | No |  | 7,943,907 | 8,653 | 1.00 (ref.) |  | 6,684,649 | 5,259 | 1.00 (ref.) |  | 7,170,300 | 6,007 | 1.00 (ref.) |
|  | Yes |  | 93,262 | 177 | **2.08 (1.78-2.42)** |  | 61,962 | 110 | **2.48 (2.03-3.03)** |  | 62,520 | 98 | **2.03 (1.64-2.52)** |

^1^ ICD10 F32-39; ^2^ ICD10 F40-42; ^3^ ICD10 F43-49; ^4^ ICD10 F32-49; ^5^ATC N06AB; ^6^ ATC N06AA; ^7^ATC N06AX+ N06AF + N06AG

**HR**=Propensity score weighted hazard ratio; **Ref**: Reference (unexposed) group. **TCA**: tricyclic antidepressant.

Significant associations are bolded.

**Table S7**: Propensity score weighted hazard ratios (95% CI) of emotional outcomes in children according to maternal exposure to SSRI (N06AB) in utero, excluding women who filled any antipsychotic medication (ATCN05A) during pregnancy (30 days before conception to birth date).

|  |  |  | | |  |
| --- | --- | --- | --- | --- | --- |
| **OUTCOME** | **Exposure** | **Person Yrs** | **Events** | **HR (95% CI)** |  |
| ***Any diagnosis or medication*** | No | 8,340,281 | 31,395 | 1.00 (ref.) |  |
|  | Yes | 124,935 | 807 | **1.54 (1.43-1.66)** |  |
| ***Diagnoses*** |  |  |  |  |  |
| Any depressive disorder^1^ | No | 8,439,792 | 5,553 | 1.00 (ref.) |  |
|  | Yes | 127,536 | 112 | **1.51 (1.24-1.83)** |  |
| Any anxiety disorder ^2^ | No | 8,426,294 | 8,786 | 1.00 (ref.) |  |
|  | Yes | 127,167 | 213 | **1.55 (1.35-1.80)** |  |
| Adjustment disorder ^3^ | No | 8,394,176 | 17,467 | 1.00 (ref.) |  |
|  | Yes | 126,458 | 414 | **1.28 (1.16-1.43)** |  |
| Any diagnosis | No | 8,352,553 | 27,989 | 1.00 (ref.) |  |
|  | Yes | 125,202 | 734 | **1.51 (1.40-1.63)** |  |
| ***Medications*** |  |  |  |  |  |
| Any SSRI^4^ | No | 8,433,312 | 7,689 | 1.00 (ref.) |  |
|  | Yes | 127,289 | 200 | **2.11 (1.82-2.45)** |  |
| Any TCA^5^ | No | 8,451,363 | 1,263 | 1.00 (ref.) |  |
|  | Yes | 127,854 | 19 | 1.13 (0.70-1.81) |  |
| Other antidepressants ^6^ | No | 8,452,773 | 1,699 | 1.00 (ref.) |  |
|  | Yes | 127,832 | 32 | **1.68 (1.16-2.42)** |  |
| Any medication | No | 8,427,156 | 9,534 | 1.00 (ref.) |  |
|  | Yes | 127,214 | 225 | **1.88 (1.64-2.17)** |  |

^1^ ICD10 F32-39; ^2^ ICD10 F40-42; ^3^ ICD10 F43-49; ^4^ ICD10 F32-49; ^5^ATC N06AB; ^6^ ATC N06AA; ^7^ATC N06AX+ N06AF + N06AG

**HR**=Propensity score weighted hazard ratio; **Ref**: Reference (unexposed) group. **TCA**: tricyclic antidepressant.

Significant associations are bolded.

**Table S8**: Propensity score weighted hazard ratios (95% CI) of emotional outcomes in children according to maternal exposure to SSRI (N06AB) (defined by >1 filled prescription in pregnancy) in utero (1997-2015)

|  |  |  | | |  |
| --- | --- | --- | --- | --- | --- |
| **OUTCOME** | **Exposure** | **Person Yrs** | **Events** | **HR (95% CI)** |  |
| ***Any diagnosis or medication*** | No | 7,642,123 | 27,558 | 1.00 (ref.) |  |
|  | Yes | 87,018 | 570 | **1.56 (1.43-1.71)** |  |
| ***Diagnoses*** |  |  |  |  |  |
| Any depressive disorder^1^ | No | 7,727,980 | 4,667 | 1.00 (ref.) |  |
|  | Yes | 88,817 | 73 | **1.45 (1.14-1.86)** |  |
| Any anxiety disorder ^2^ | No | 7,715,732 | 7,759 | 1.00 (ref.) |  |
|  | Yes | 88,579 | 143 | **1.48 (1.25-1.77)** |  |
| Adjustment disorder ^3^ | No | 7,688,083 | 15,478 | 1.00 (ref.) |  |
|  | Yes | 88,085 | 283 | 1.25 (1.11-1.42) |  |
| Any diagnosis | No | 7,651,975 | 24,828 | 1.00 (ref.) |  |
|  | Yes | 87,223 | 510 | **1.49 (1.36-1.64)** |  |
| ***Medications*** |  |  |  |  |  |
| Any SSRI^4^ | No | 7,723,142 | 6,279 | 1.00 (ref.) |  |
|  | Yes | 88,627 | 141 | **2.26 (1.89-2.70)** |  |
| Any TCA^5^ | No | 7,737,352 | 1,079 | 1.00 (ref.) |  |
|  | Yes | 89,020 | 13 | 1.16 (0.66-2.05) |  |
| Other antidepressants ^6^ | No | 7,738,896 | 1,220 | 1.00 (ref.) |  |
|  | Yes | 89,016 | 13 | 1.13 (0.64-1.99) |  |
| Any medication | No | 7,718,135 | 7,781 | 1.00 (ref.) |  |
|  | Yes | 88,593 | 156 | **2.00 (1.69-2.36)** |  |

^1^ ICD10 F32-39; ^2^ ICD10 F40-42; ^3^ ICD10 F43-49; ^4^ ICD10 F32-49; ^5^ATC N06AB; ^6^ ATC N06AA; ^7^ATC N06AX+ N06AF + N06AG

**HR**=Propensity score weighted hazard ratio; **Ref**: Reference (unexposed) group. **TCA**: tricyclic antidepressant.

Significant associations are bolded.

**Table S9**: Propensity score weighted hazard ratios (95% CI) of emotional outcomes in children according to maternal exposure to SSRI (N06AB) using complete case data.

|  |  |  | **Complete Cases** |  |  |  | **Complete Case ± BMI** |  |  |
| --- | --- | --- | --- | --- | --- | --- | --- | --- | --- |
| **OUTCOME** | **Exposure** |  | **Person Yrs** | **Events** | **HR (95% CI)** |  | **Person Yrs** | **Events** | **HR (95% CI)** |
| ***Any diagnosis or medication*** | No |  | 3,561,096 | 6,980 | 1.00 (ref.) |  | 7,215,917 | 25,550 | 1.00 (ref.) |
|  | Yes |  | 86,355 | 371 | **1.69 (1.51-1.88)** |  | 120,102 | 729 | **1.56 (1.45-1.69)** |
| ***Diagnoses*** |  |  |  |  |  |  |  |  |  |
| Any depressive disorder^1^ | No |  | 3,580,143 | 439 | 1.00 (ref.) |  | 7,293,395 | 4,239 | 1.00 (ref.) |
|  | Yes |  | 87,421 | 21 | **1.62 (1.03-2.57)** |  | 122,411 | 93 | **1.44 (1.16-1.77)** |
| Any anxiety disorder ^2^ | No |  | 3,576,465 | 1,779 | 1.00 (ref.) |  | 7,281,497 | 7,273 | 1.00 (ref.) |
|  | Yes |  | 87,248 | 71 | **1.47 (1.15-1.88)** |  | 122,030 | 191 | **1.57 (1.36-1.83)** |
| Adjustment disorder ^3^ | No |  | 3,570,009 | 3,938 | 1.00 (ref.) |  | 7,256,706 | 14,527 | 1.00 (ref.) |
|  | Yes |  | 86,894 | 192 | **1.44 (1.24-1.67)** |  | 121,405 | 379 | **1.31 (1.18-1.46)** |
| Any diagnosis | No |  | 3,562,611 | 6,516 | 1.00 (ref.) |  | 7,224,027 | 23,242 | 1.00 (ref.) |
|  | Yes |  | 86,402 | 350 | **1.68 (1.50-1.88)** |  | 120,324 | 664 | **1.52 (1.41-1.65)** |
| ***Medications*** |  |  |  |  |  |  |  |  |  |
| Any SSRI^4^ | No |  | 3,579,863 | 543 | 1.00 (ref.) |  | 7,290,300 | 5,454 | 1.00 (ref.) |
|  | Yes |  | 87,378 | 38 | **2.76 (1.93-3.94)** |  | 122,209 | 162 | **2.12 (1.80-2.50)** |
| Any TCA^5^ | No |  | 3,579,767 | 342 | 1.00 (ref.) |  | 7,301,094 | 992 | 1.00 (ref.) |
|  | Yes |  | 87,443 | 7 | 0.82 (0.38-1.76) |  | 122,634 | 16 | 1.07 (0.65-1.77) |
| Other antidepressants ^6^ | No |  | 3,580,817 | 22 | 1.00 (ref.) |  | 7,303,277 | 825 | 1.00 (ref.) |
|  | Yes |  | 87,456 | <5 | 2.40 (0.31-18.83) |  | 122,628 | 17 | 1.62 (0.99-2.65) |
| Any medication | No |  | 3,578,686 | 897 | 1.00 (ref.) |  | 7,285,932 | 6,751 | 1.00 (ref.) |
|  | Yes |  | 87,364 | 45 | **1.99 (1.45-2.74)** |  | 122,144 | 181 | **1.89 (1.62-2.21)** |

^1^ ICD10 F32-39; ^2^ ICD10 F40-42; ^3^ ICD10 F43-49; ^4^ ICD10 F32-49; ^5^ATC N06AB; ^6^ ATC N06AA; ^7^ATC N06AX+ N06AF + N06AG

**HR**=Propensity score weighted hazard ratio; **Ref**: Reference (unexposed) group. **TCA**: tricyclic antidepressant.

Significant associations are bolded.

Complete Case Dataset (left) only includes mothers with no missing data. The complete case **±** BMI dataset is agnostic to missingness of BMI but requires all other variables to be present. The reason for conducting the analysis with and without BMI requirements was that prior to 2003, reporting of BMI into the registers was not required, and thus most pregnancies prior to 2003 did not have BMI. We considered this to act as a missing-at-random variable.

**Table S6:** Propensity score weighted hazard ratios (95% CI) of emotional outcomes in children according to maternal exposure to SSRI (N06AB) in utero stratified by trimester (1997-2015)

|  |  |  | **First Trimester Exposure** | | |  | **Second Trimester Exposure** | | |  | **Third Trimester Exposure** | | |
| --- | --- | --- | --- | --- | --- | --- | --- | --- | --- | --- | --- | --- | --- |
| **OUTCOME** | **Exposure** |  | **Person Yrs** | **Events** | **HR (95% CI)** |  | **Person Yrs** | **Events** | **HR (95% CI)** |  | **Person Yrs** | **Events** | **HR (95% CI)** |
| ***Any diagnosis or medication*** | No |  | 7,862,330 | 29,482 | 1.00 (ref.) |  | 6,624,341 | 21,779 | 1.00 (ref.) |  | 7,104,670 | 23,764 | 1.00 (ref.) |
|  | Yes |  | 91,546 | 621 | **1.64 (1.52-1.78**) |  | 60,807 | 409 | **1.74 (1.56-1.93)** |  | 61,450 | 391 | **1.61 (1.44-1.79)** |
| ***Diagnoses*** |  |  |  |  |  |  |  |  |  |  |  |  |  |
| Any depressive disorder^1^ | No |  | 7,955,207 | 5,083 | 1.00 (ref.) |  | 6,691,179 | 3,208 | 1.00 (ref.) |  | 7,177,778 | 3,628 | 1.00 (ref.) |
|  | Yes |  | 93,569 | 82 | **1.53 (1.22-1.92)** |  | 62,151 | 49 | **1.66 (1.24-2.23)** |  | 62,673 | 48 | **1.53 (1.13-2.08)** |
| Any anxiety disorder ^2^ | No |  | 7,942,312 | 8,260 | 1.00 (ref.) |  | 6,680,899 | 6,052 | 1.00 (ref.) |  | 7,166,545 | 6,678 | 1.00 (ref.) |
|  | Yes |  | 93,250 | 168 | **1.72 (1.47-2.01)** |  | 61,918 | 108 | **1.78 (1.45-2.17)** |  | 62,454 | 103 | **1.61 (1.31-1.98)** |
| Adjustment disorder ^3^ | No |  | 7,912,160 | 16,498 | 1.00 (ref.) |  | 6,658,461 | 12,508 | 1.00 (ref.) |  | 7,142,534 | 13,533 | 1.00 (ref.) |
|  | Yes |  | 92,748 | 317 | 1.36 (1.21-1.52) |  | 61,626 | 194 | 1.33 (1.14-1.54) |  | 62,206 | 188 | 1.22 (1.05-1.42) |
| Any diagnosis | No |  | 7,873,387 | 26,410 | 1.00 (ref.) |  | 6,630,981 | 19,952 | 1.00 (ref.) |  | 7,112,166 | 21,675 | 1.00 (ref.) |
|  | Yes |  | 91,790 | 560 | **1.60 (1.47-1.74)** |  | 60,965 | 364 | **1.64 (1.47-1.83)** |  | 61,579 | 354 | **1.55 (1.39-1.73)** |
| ***Medications*** |  |  |  |  |  |  |  |  |  |  |  |  |  |
| Any SSRI^4^ | No |  | 7,949,526 | 6,975 | 1.00 (ref.) |  | 6,688,357 | 4,171 | 1.00 (ref.) |  | 7,174,327 | 4,807 | 1.00 (ref.) |
|  | Yes |  | 93,328 | 158 | **2.34 (1.99-2.76)** |  | 61,979 | 99 | **2.87 (2.32-3.54)** |  | 62,528 | 92 | **2.43 (1.94-3.04)** |
| Any TCA^5^ | No |  | 7,965,577 | 1,174 | 1.00 (ref.) |  | 6,696,793 | 816 | 1.00 (ref.) |  | 7,184,447 | 904 | 1.00 (ref.) |
|  | Yes |  | 93,779 | 13 | 1.11 (0.64-1.93) |  | 62,290 | 8 | 0.94 (0.45-1.95) |  | 62,807 | <5 | 0.42 (0.16-1.11) |
| Other antidepressants ^6^ | No |  | 7,967,083 | 1,461 | 1.00 (ref.) |  | 6,698,300 | 675 | 1.00 (ref.) |  | 7,186,056 | 782 | 1.00 (ref.) |
|  | Yes |  | 93,769 | 19 | 1.37 (0.86-2.17) |  | 62,279 | 12 | 2.27 (1.24-4.16) |  | 62,791 | 9 | 1.75 (0.89-3.46) |
| Any medication | No |  | 7,943,907 | 8,653 | 1.00 (ref.) |  | 6,684,649 | 5,259 | 1.00 (ref.) |  | 7,170,300 | 6,007 | 1.00 (ref.) |
|  | Yes |  | 93,262 | 177 | **2.08 (1.78-2.42)** |  | 61,962 | 110 | **2.48 (2.03-3.03)** |  | 62,520 | 98 | **2.03 (1.64-2.52)** |

^1^ ICD10 F32-39; ^2^ ICD10 F40-42; ^3^ ICD10 F43-49; ^4^ ICD10 F32-49; ^5^ATC N06AB; ^6^ ATC N06AA; ^7^ATC N06AX+ N06AF + N06AG

**HR**=Propensity score weighted hazard ratio; **Ref**: Reference (unexposed) group. **TCA**: tricyclic antidepressant.

Significant associations are bolded.

**Table S10**: Propensity score weighted hazard ratios (95% CI) of emotional outcomes in children according to paternal exposure to SSRI during the index pregnancy (1997-2015)

|  |  |  | **Paternal SSRI Use**  *(Agnostic to maternal psychiatric diagnosis or medication use)* | | |  | **Paternal SSRI Use**  *(In the absence of maternal psychiatric diagnosis or medication use)* | | |
| --- | --- | --- | --- | --- | --- | --- | --- | --- | --- |
| **OUTCOME** | **Exposure** |  | **Person Yrs** | **Events** | **HR (95% CI)** |  | **Person Yrs** | **Events** | **HR (95% CI)** |
| ***Any diagnosis or medication*** | No |  | 9,955,832 | 39,919 | 1.00 (ref.) |  | 9,658,707 | 38,119 | 1.00 (ref.) |
|  | Yes |  | 128,005 | 700 | **1.48 (1.37-1.60)** |  | **116,991** | **623** | **1.46 (1.34-1.58)** |
| ***Diagnoses*** |  |  |  |  |  |  |  |  |  |
| Any depressive disorder^1^ | No |  | 10,082,831 | 7,665 | 1.00 (ref.) |  | 9,779,901 | 7,376 | 1.00 (ref.) |
|  | Yes |  | 130,233 | 107 | **1.40 (1.16-1.70)** |  | **118,979** | **100** | **1.42 (1.16-1.73)** |
| Any anxiety disorder ^2^ | No |  | 10,066,930 | 11,190 | 1.00 (ref.) |  | 9,764,744 | 10,719 | 1.00 (ref.) |
|  | Yes |  | 129,769 | 226 | **1.80 (1.57-2.05)** |  | **118,583** | **204** | **1.77 (1.53-2.03)** |
| Adjustment disorder ^3^ | No |  | 10,028,086 | 21,549 | 1.00 (ref.) |  | 9,727,798 | 20,550 | 1.00 (ref.) |
|  | Yes |  | 129,298 | 369 | **1.35 (1.21-1.50)** |  | **118,142** | **326** | **1.32 (1.18-1.47)** |
| Any diagnosis | No |  | 9,973,023 | 35,069 | 1.00 (ref.) |  | 9,675,272 | 33,436 | 1.00 (ref.) |
|  | Yes |  | 128,253 | 632 | **1.48 (1.36-1.60)** |  | **117,236** | **559** | **1.45 (1.33-1.58)** |
| ***Medications*** |  |  |  |  |  |  |  |  |  |
| Any SSRI^4^ | No |  | 10,072,935 | 11,029 | 1.00 (ref.) |  | 9,770,492 | 10,606 | 1.00 (ref.) |
|  | Yes |  | 129,917 | 194 | **1.89 (1.63-2.19)** |  | **118,677** | **180** | **1.89 (1.63-2.20)** |
| Any TCA^5^ | No |  | 10,099,960 | 1,656 | 1.00 (ref.) |  | 9,796,326 | 1,613 | 1.00 (ref.) |
|  | Yes |  | 130,465 | 21 | 1.15 (0.74-1.77) |  | 119,196 | 20 | 1.17 (0.75-1.83) |
| Other antidepressants ^6^ | No |  | 10,101,511 | 2,411 | 1.00 (ref.) |  | 9,797,917 | 2,338 | 1.00 (ref.) |
|  | Yes |  | 130,487 | 32 | **1.42 (1.00-2.02)** |  | **119,218** | **31** | **1.45 (1.02-2.08)** |
| Any medication | No |  | 10,064,879 | 13,465 | 1.00 (ref.) |  | 9,762,648 | 12,964 | 1.00 (ref.) |
|  | Yes |  | 129,828 | 223 | **1.73 (1.51-1.98)** |  | **118,589** | **207** | **1.73 (1.51-2.00)** |

^1^ ICD10 F32-39; ^2^ ICD10 F40-42; ^3^ ICD10 F43-49; ^4^ ICD10 F32-49; ^5^ATC N06AB; ^6^ ATC N06AA; ^7^ATC N06AX+ N06AF + N06AG

**HR**=Propensity score weighted hazard ratio; **Ref**: Reference (unexposed) group. **TCA**: tricyclic antidepressant.

Significant associations are bolded.

**Table S11**: Propensity score weighted hazard ratios (95% CI) of emotional outcomes in children according to postpartum maternal exposure to SSRI (N06AB) from birth to 2 years postpartum, follow-up started 2 years postpartum (1997-2015)

|  |  |  | | |  |
| --- | --- | --- | --- | --- | --- |
| **OUTCOME** | **Exposure** | **Person Yrs** | **Events** | **HR (95% CI)** |  |
| ***Any diagnosis or medication*** | No | 10,318,329 | 40,007 | 1.00 (ref.) |  |
|  | Yes | 315,883 | 2,009 | **1.42 (1.35-1.49)** |  |
| ***Diagnoses*** |  |  |  |  |  |
| Any depressive disorder^1^ | No | 10,446,195 | 7,725 | 1.00 (ref.) |  |
|  | Yes | 322,578 | 383 | **1.60 (1.43-1.79)** |  |
| Any anxiety disorder ^2^ | No | 10,430,143 | 11,279 | 1.00 (ref.) |  |
|  | Yes | 321,761 | 567 | **1.52 (1.39-1.67)** |  |
| Adjustment disorder ^3^ | No | 10,390,558 | 21,573 | 1.00 (ref.) |  |
|  | Yes | 319,615 | 1,122 | **1.33 (1.24-1.42)** |  |
| Any diagnosis | No | 10,336,077 | 34,986 | 1.00 (ref.) |  |
|  | Yes | 316,751 | 1,797 | **1.39 (1.32-1.47)** |  |
| ***Medications*** |  |  |  |  |  |
| Any SSRI^4^ | No | 10,436,253 | 11,111 | 1.00 (ref.) |  |
|  | Yes | 321,993 | 554 | **1.77 (1.61-1.94)** |  |
| Any TCA^5^ | No | 10,463,152 | 1,749 | 1.00 (ref.) |  |
|  | Yes | 323,463 | 62 | 1.25 (0.95-1.64) |  |
| Other antidepressants ^6^ | No | 10,464,643 | 2,621 | 1.00 (ref.) |  |
|  | Yes | 323,447 | 124 | **1.83 (1.51-2.21)** |  |
| Any medication | No | 10,427,594 | 13,725 | 1.00 (ref.) |  |
|  | Yes | 321,617 | 657 | **1.69 (1.56-1.84)** |  |

^1^ ICD10 F32-39; ^2^ ICD10 F40-42; ^3^ ICD10 F43-49; ^4^ ICD10 F32-49; ^5^ATC N06AB; ^6^ ATC N06AA; ^7^ATC N06AX+ N06AF + N06AG

**HR**=Propensity score weighted hazard ratio; **Ref**: Reference (unexposed) group. **TCA**: tricyclic antidepressant.

Significant associations are bolded. Note, women who used any SSRI during pregnancy were excluded from this analysis.

**Table S12:** Combined model showing individual and combined effects of maternal and paternal SSRI use during pregnancy.

|  |  | **OUTCOME** | **Person Yrs** | **Events** | **HR-Crude** | **HR-Adjusted** |
| --- | --- | --- | --- | --- | --- | --- |
|  | | |  |  |  |  |
|  |  | ***Any Diagnosis or Medication*** |  |  |  |  |
|  | *0* | *Reference (No Exposure)* | 10,919,007 | 42,125 | 1.00 (ref.) | 1.00 (ref.) |
|  | *1* | Maternal Prenatal only | 211,778 | 1,148 | **1.99 (1.88-2.11)** | **1.34 (1.26-1.42)** |
|  | *2* | Maternal Postnatal only | 349,210 | 2,252 | **2.02 (1.94-2.11)** | **1.48 (1.41-1.55)** |
|  | *3* | Father prenatal only | 183,461 | 857 | **1.62 (1.51-1.73)** | **1.19 (1.11-1.28)** |
|  | *4* | Maternal Pre and Postnatal | 169,876 | 1,104 | **2.66 (2.50-2.82)** | **1.58 (1.48-1.69)** |
|  | *5* | Maternal and Paternal Prenatal | 12,120 | 71 | **2.91 (2.30-3.67)** | **1.53 (1.21-1.93)** |
|  | *6* | Paternal Pre + Maternal Post | 13,841 | 80 | **2.38 (1.91-2.96)** | **1.31 (1.05-1.63)** |
|  | *7* | ALL | 11,852 | 88 | **3.77 (3.05-4.65)** | **1.62 (1.30-2.01)** |
|  |  |  |  |  |  |  |
|  |  | ***Depressive disorders (ICD10 F32-39)*** |  |  |  |  |
|  | *0* | *Reference* | 11,054,100 | 8,310 | 1.00 (ref.) | 1.00 (ref.) |
|  | *1* | Maternal Prenatal only | 215,415 | 182 | **1.82 (1.57-2.11)** | **1.38 (1.19-1.60)** |
|  | *2* | Maternal Postnatal only | 356,817 | 416 | **2.02 (1.83-2.23)** | **1.63 (1.47-1.80)** |
|  | *3* | Father prenatal only | 186,309 | 118 | **1.26 (1.05-1.51)** | 1.01 (0.84-1.23) |
|  | *4* | Maternal Pre and Postnatal | 173,481 | 161 | **2.39 (2.05-2.80)** | **1.62 (1.37-1.92)** |
|  | *5* | Maternal and Paternal Prenatal | 12,299 | 8 | **2.29 (1.14-4.57)** | 1.48 (0.73-2.99) |
|  | *6* | Paternal Pre + Maternal Post | 14,084 | 10 | **1.94 (1.05-3.58)** | 1.28 (0.68-2.40) |
|  | *7* | ALL | 12,135 | 13 | **3.88 (2.26-6.66)** | **2.12 (1.22-3.67)** |
|  |  |  |  |  |  |  |
|  |  | ***Anxiety disorders*** |  |  |  |  |
|  | *0* | *Reference (No Exposure)* | 11,037,774 | 11,820 | 1.00 (ref.) | 1.00 (ref.) |
|  | *1* | Maternal Prenatal only | 214,968 | 288 | **1.79 (1.59-2.01)** | **1.29 (1.14-1.45)** |
|  | *2* | Maternal Postnatal only | 355,909 | 614 | **1.94 (1.78-2.10)** | **1.54 (1.42-1.67)** |
|  | *3* | Father prenatal only | 185,810 | 250 | **1.70 (1.50-1.92)** | **1.29 (1.13-1.48)** |
|  | *4* | Maternal Pre and Postnatal | 172,948 | 300 | **2.58 (2.30-2.90)** | **1.65 (1.46-1.87)** |
|  | *5* | Maternal and Paternal Prenatal | 12,262 | 18 | **2.75 (1.73-4.38)** | **1.63 (1.01-2.61)** |
|  | *6* | Paternal Pre + Maternal Post | 14,051 | 21 | **2.26 (1.48-3.45)** | 1.41 (0.91-2.17) |
|  | *7* | ALL | 12,087 | 28 | **4.33 (3.00-6.26)** | **2.18 (1.49-3.19)** |
|  |  |  |  |  |  |  |
|  |  | ***Adjustment disorders*** |  |  |  |  |
|  | *0* | *Reference (No Exposure)* | 10,996,541 | 22,550 | 1.00 (ref.) | 1.00 (ref.) |
|  | *1* | Maternal Prenatal only | 213,840 | 627 | **2.00 (1.84-2.16)** | **1.21 (1.11-1.32)** |
|  | *2* | Maternal Postnatal only | 353,317 | 1,280 | **2.10 (1.98-2.22)** | **1.39 (1.31-1.47)** |
|  | *3* | Father prenatal only | 184,963 | 455 | **1.58 (1.44-1.74)** | 1.08 (0.98-1.19) |
|  | *4* | Maternal Pre and Postnatal | 172,055 | 562 | **2.46 (2.26-2.67)** | **1.30 (1.19-1.43)** |
|  | *5* | Maternal and Paternal Prenatal | 12,197 | 44 | **3.34 (2.48-4.50)** | **1.47 (1.09-1.98)** |
|  | *6* | Paternal Pre + Maternal Post | 13,957 | 47 | **2.58 (1.94-3.43)** | 1.19 (0.89-1.59) |
|  | *7* | ALL | 12,009 | 49 | **3.81 (2.85-5.08)** | **1.35 (1.00-1.81)** |
|  |  |  |  |  |  |  |
|  |  | ***Any diagnosis*** |  |  |  |  |
|  | *0* | *Reference (No Exposure)* | 10,938,202 | 36,704 | 1.00 (ref.) | 1.00 (ref.) |
|  | *1* | Maternal Prenatal only | 212,224 | 1,028 | **2.01 (1.88-2.14)** | **1.31 (1.22-1.40)** |
|  | *2* | Maternal Postnatal only | 350,160 | 2,026 | **2.05 (1.96-2.15)** | **1.46 (1.39-1.53)** |
|  | *3* | Father prenatal only | 183,824 | 767 | **1.63 (1.52-1.76)** | **1.18 (1.09-1.28)** |
|  | *4* | Maternal Pre and Postnatal | 170,297 | 987 | **2.65 (2.49-2.83)** | **1.53 (1.42-1.64)** |
|  | *5* | Maternal and Paternal Prenatal | 12,122 | 67 | **3.08 (2.42-3.92)** | **1.55 (1.22-1.98)** |
|  | *6* | Paternal Pre + Maternal Post | 13,861 | 72 | **2.38 (1.89-3.00)** | **1.26 (1.00-1.59)** |
|  | *7* | ALL | 11,854 | 87 | **4.17 (3.38-5.16)** | **1.71 (1.38-2.13)** |
|  |  |  |  |  |  |  |
|  |  | ***SSRI (ATC N06AB)*** |  |  |  |  |
|  | *0* | *Reference (No Exposure)* | 11,043,313 | 12,010 | 1.00 (ref.) | 1.00 (ref.) |
|  | *1* | Maternal Prenatal only | 215,091 | 274 | **1.97 (1.74-2.22)** | **1.55 (1.37-1.75)** |
|  | *2* | Maternal Postnatal only | 356,143 | 600 | **2.09 (1.93-2.27)** | **1.77 (1.63-1.93)** |
|  | *3* | Father prenatal only | 185,979 | 205 | **1.57 (1.36-1.80)** | **1.28 (1.10-1.48)** |
|  | *4* | Maternal Pre and Postnatal | 173,020 | 298 | **3.22 (2.87-3.62)** | **2.32 (2.04-2.64)** |
|  | *5* | Maternal and Paternal Prenatal | 12,292 | 11 | **2.24 (1.24-4.06)** | 1.56 (0.85-2.87) |
|  | *6* | Paternal Pre + Maternal Post | 14,066 | 18 | **2.52 (1.59-4.00)** | **1.76 (1.10-2.80)** |
|  | *7* | ALL | 12,139 | 13 | **2.73 (1.61-4.61)** | 1.54 (0.90-2.63) |
|  |  |  |  |  |  |  |
|  |  | ***TCA*** |  |  |  |  |
|  | *0* | *Reference (No Exposure)* | 11,072,903 | 1,851 | 1.00 (ref.) | 1.00 (ref.) |
|  | *1* | Maternal Prenatal only | 215,825 | 41 | **1.54 (1.13-2.10)** | 1.37 (0.99-1.90) |
|  | *2* | Maternal Postnatal only | 357,766 | 69 | **1.37 (1.08-1.74)** | 1.26 (0.98-1.61) |
|  | *3* | Father prenatal only | 186,538 | 27 | 1.12 (0.77-1.64) | 0.96 (0.64-1.43) |
|  | *4* | Maternal Pre and Postnatal | 173,889 | 29 | **1.47 (1.02-2.13)** | 1.26 (0.86-1.86) |
|  | *5* | Maternal and Paternal Prenatal | 12,316 | <5 | 1.68 (0.42-6.70) | 1.37 (0.34-5.56) |
|  | *6* | Paternal Pre + Maternal Post | 14,113 | <5 | 1.28 (0.32-5.12) | 0.95 (0.24-3.83) |
|  | *7* | ALL | 12,175 | 0 | - | - |
|  |  |  |  |  |  |  |
|  |  | ***Other antidepressants*** |  |  |  |  |
|  | *0* | *Reference (No Exposure)* | 11,074,398 | 2,828 | 1.00 (ref.) | 1.00 (ref.) |
|  | *1* | Maternal Prenatal only | 215,834 | 52 | **1.87 (1.42-2.46)** | **1.41 (1.06-1.88)** |
|  | *2* | Maternal Postnatal only | 357,772 | 130 | **2.20 (1.84-2.62)** | **1.72 (1.43-2.06)** |
|  | *3* | Father prenatal only | 186,515 | 47 | **1.79 (1.34-2.38)** | 1.34 (0.98-1.82) |
|  | *4* | Maternal Pre and Postnatal | 173,866 | 48 | **2.84 (2.14-3.77)** | **1.93 (1.43-2.61)** |
|  | *5* | Maternal and Paternal Prenatal | 12,317 | <5 | 2.50 (0.62-10.09) | 1.75 (0.43-7.09) |
|  | *6* | Paternal Pre + Maternal Post | 14,098 | 6 | **5.21 (2.33-11.64)** | **3.05 (1.36-6.85)** |
|  | *7* | ALL | 12,170 | <5 | **3.72 (1.21-11.44)** | 1.81 (0.57-5.81) |
|  |  |  |  |  |  |  |
|  |  | ***Any antidepressant medication*** |  |  |  |  |
|  | *0* | *Reference (No Exposure)* | 11,034,141 | 14,769 | 1.00 (ref.) | 1.00 (ref.) |
|  | *1* | Maternal Prenatal only | 214,906 | 332 | **1.90 (1.71-2.12)** | **1.50 (1.34-1.68)** |
|  | *2* | Maternal Postnatal only | 355,740 | 711 | **2.01 (1.86-2.16)** | **1.69 (1.56-1.83)** |
|  | *3* | Father prenatal only | 185,817 | 253 | **1.55 (1.37-1.76)** | **1.26 (1.10-1.44)** |
|  | *4* | Maternal Pre and Postnatal | 172,916 | 337 | **2.88 (2.58-3.21)** | **2.08 (1.84-2.34)** |
|  | *5* | Maternal and Paternal Prenatal | 12,290 | 15 | **2.36 (1.43-3.91)** | 1.63 (0.98-2.73) |
|  | *6* | Paternal Pre + Maternal Post | 14,058 | 21 | **2.33 (1.52-3.56)** | **1.59 (1.03-2.44)** |
|  | *7* | ALL | 12,137 | 14 | **2.26 (1.36-3.75)** | 1.27 (0.76-2.12) |
|  |  |  |  |  |  |  |
|  |  |  |  |  |  |  |

^1^ ICD10 F32-39; ^2^ ICD10 F40-42; ^3^ ICD10 F43-49; ^4^ ICD10 F32-49; ^5^ATC N06AB; ^6^ ATC N06AA; ^7^ATC N06AX+ N06AF + N06AG

**HR**=Propensity score weighted hazard ratio; **Ref**: Reference (unexposed) group. **TCA**: tricyclic antidepressant. Significant associations are bolded. **Figure S1.** Failure plots on hazard rates by age for each diagnosis and prescription outcome by maternal exposure to selective serotonin reuptake inhibitor (SSRI) during pregnancy in Denmark (1997-2015).
